# Prebiotics improve frailty status in community-dwelling older individuals in a double-blind, randomized, controlled trial

**DOI:** 10.1172/JCI176507

**Published:** 2024-08-06

**Authors:** Jie Yang, Liming Hou, Anhui Wang, Lei Shang, Xin Jia, Rong Xu, Xiaoming Wang

**Affiliations:** 1 Department of Geriatrics, Xijing Hospital,; 2 Department of Epidemiology, and; 3 Department of Health Statistics, Air Force Medical University, Xi’an, China.

**Keywords:** Aging, Clinical trials, Clinical practice

## Abstract

**BACKGROUND:**

Frailty significantly affects morbidity and mortality rates in the older population (age >65 years). Age-related degenerative diseases are influenced by the intestinal microbiota. However, limited research exists on alterations in the intestinal microbiota in frail older individuals, and the effectiveness of prebiotic intervention for treating frailty remains uncertain.

**OBJECTIVE:**

We sought to examine the biological characteristics of the intestinal microbiome in frail older individuals and assess changes in both frailty status and gut microbiota following intervention with a prebiotic blend consisting of inulin and oligofructose.

**METHODS:**

The study consisted of 3 components: an observational analysis with a sample size of 1,693, a cross-sectional analysis (*n* = 300), and a multicenter double-blind, randomized, placebo-controlled trial (*n* = 200). Body composition, commonly used scales, biochemical markers, intestinal microbiota, and metabolites were examined in 3 groups of older individuals (nonfrail, prefrail, and frail). Subsequently, changes in these indicators were reevaluated after a 3-month intervention using the prebiotic mixture for the prefrail and frail groups.

**RESULTS:**

The intervention utilizing a combination of prebiotics significantly improved frailty and renal function among the older population, leading to notable increases in protein levels, body fat percentage, walking speed, and grip strength. Additionally, it stimulated an elevation in gut probiotic count and induced alterations in microbial metabolite expression levels as well as corresponding metabolic pathways.

**CONCLUSIONS:**

The findings suggest a potential link between changes in the gut microbiota and frailty in older adults. Prebiotics have the potential to modify the gut microbiota and metabolome, resulting in improved frailty status and prevention of its occurrence.

**TRIAL REGISTRATION:**

ClinicalTrials.gov NCT03995342.

## Abstract 2

The intestinal microbiota may be associated with frailty in elderly individuals and that prebiotics could modulate the intestinal microbiota and metabolome.

## Introduction

Approximately 21.3% of the global population will be 60 years of age or older by 2050, with the global life expectancy at birth increasing from 66.8 years in 2000 to 73.3 years in 2019. However, a healthy, disease-free lifespan (health span) has not increased as much as lifespan, as advanced age is a major risk factor for several diseases, including cancer, cardiovascular disease, and neurodegenerative disease, and the increasing proportion of unhealthy older people is posing a global challenge to society (1, 2). Frailty, characterized by exhaustion, chronic malnutrition, decreased physical activity, and mobility disorders affects more than 13.6% of older adults globally (3, 4) This syndrome represents the most challenging aspect of population aging, as it substantially increases the risk of falls, disability, long-term care, and even death among older people. In addition to heightened vulnerability and individual dependence, the proposed mechanisms underlying frailty include chronic inflammation, immune system disorders, and mitochondrial DNA abnormalities (5, 6). At present, effective prevention and treatment measures for frailty are lacking, and the common intervention measures, including exercise, nutritional supplementation, rational drug treatment, comorbidity management, and psychological intervention, are still being explored (5, 7).

The intestinal microbiota of the fetus begins to build up in the womb and undergo rapid modification during the first 3 years of life, followed by relative stabilization until 65 years of age. Then, the composition of the microbiota begins to change gradually and accelerates after 70 years of age, with increased variability, reduced biodiversity, and pathogen colonization (8, 9). Microorganisms are dynamic, adaptable, and plastic, and their composition shows great individual differences; therefore, they are also known as “the second genome,” which affects health status. Growing evidence indicates a relationship between the intestinal microbiota and a broad range of diseases, including obesity, type 2 diabetes, and chronic and geriatric syndromes (10, 11). Previous research conducted in frail older adults has suggested that altered intestinal permeability and changes in the intestinal muscle axis and the gut-brain axis may be key factors associated with the pathophysiology of frailty (12, 13). In contrast, however, 2 studies involving community-dwelling adults showed that frailty was negatively associated with intestinal microbiota diversity (14, 15).

In the past decade, understanding of the intestinal microbiota has rapidly increased, accompanied by increased interest in prebiotics as a means to modulate the intestinal microbiota, as they have been defined as nondigestible food ingredients that confer multiple health benefits by selectively stimulating the growth and/or activity of one or a limited number of bacteria in the colon when administered in adequate amounts (16, 17). Paul et al. pointed out in 2015 that, because the microbiota may modulate aging-related changes in innate immunity, sarcopenia, and cognitive function, all of which are elements of frailty, the potential for the intestinal microbiota to affect health has a particular relevance for older individuals (18). In recent years, 5 review articles have discussed the link between physical frailty and intestinal microbiota (19–23) and concluded that, given the lack of targeted studies and the influence of a large number of covariates, including diet, exercise, multimorbidity, and polypharmacy on both microbiota composition and physical function in older individuals, the causal link between microbiota and frailty is still uncertain. Moreover, 2 clinical studies have investigated the effects of prebiotics on frail older people. Cristina et al. reported an improvement in several frailty parameters (exhaustion and handgrip strength) through the use of prebiotics, but these studies focused only on observing frailty status and lacked information on whether the changes in frailty status are related to the gut microbiota and its metabolites (24, 25). Several reviews of the current scientific literature have shown that the use of prebiotics is a cost-effective and widely available intervention that may improve the homeostasis of the intestinal microbiota and prevent frailty and unhealthy aging, but no direct conclusions can be drawn regarding the efficacy of these measures (26–29). In addition, all of the previous studies have overlooked the large number of older people in the prefrail stage, considering that these individuals constitute a significant proportion of the community (as confirmed in 2021 by Nicola Veronese, who estimated that the prevalence of prefrailty was 36.4%; ref. 30).

In summary, the relationship between the intestinal microbiota and frailty status remains a very promising area of research for the future. Therefore, in this study, for the first time, we aimed not only to observe differences in the intestinal microbiota and metabolome between frail and prefrail older individuals but also to explore the effects of a 12-week prebiotic intervention on older people with different frailty states and to determine whether prebiotic supplementation could modulate the intestinal microbiota and metabolome and improve frailty status in this population.

## Results

### Frailty status screening in the older population

A total of 1,693 older individuals (>65 years of age) were screened for frailty status (Table 1): 703 were in the nonfrail group (N), 705 were in the prefrail group (P), and 285 were in the frail group (F). The overall prevalence of prefrailty was 41.6% (53.4% for men and 33.0% for women, *P* < 0.001), and the prevalence of frailty was 16.8% (16.7% for men and 16.9% for women, *P* = 0.892). The mean ages of the 3 groups were significantly different (*P* < 0.001), and the degree of frailty was positively correlated with age. With increasing frailty severity, the number of older people who experienced weight loss and physical decline increased, accompanied by slower walking speed, decreased grip strength, and increased exhaustion score, and there were significant differences between the groups (*P* < 0.01).

### Differences between groups with different frailty states among older people

#### Sociodemography and general health status.

The results showed that there were statistically significant differences (*P* < 0.05) among the 3 groups (*n* = 300 total) of older people in terms of age, number of children, number of comorbidities, surgical history, exercise frequency, degree of exhaustion, and whether acute events occurred in the past year. A high frequency of exercise was a protective factor and negatively correlated with frailty. There were no statistically significant differences (*P* > 0.05) with regard to sex, BMI, occupation, education level, marital status, income status, living situation, tobacco consumption, or alcohol consumption (Table 2).

#### Differences in frailty indicators, scale scores, body composition, and food and main nutrient intake among groups with different frailty states.

Compared with the individuals in group F, the scores for daily living ability (ADL) in groups N and P were higher, indicating that the self-care ability of frail older individuals was poorer (*P* = 0.019 and *P* = 0.049). The sleep quality score (PSQI) for group F was higher than that of group N, indicating that the sleep quality of frail older individuals was poor (*P* < 0.001). There was no significant difference between the other scales (*P* > 0.05). There were no significant differences in body weight, BMI, body fat percentage, muscle mass, estimated bone mass, basal metabolism, or body moisture rate among the 3 groups (*P* > 0.05), except for significant differences in visceral fat (*P* = 0.009). Visceral fat in group F was significantly lower than that in group N (*P* = 0.004). There was no difference among the other groups (N vs. P, *Z* = 5.0, *P* = 0.025; P vs. F, *Z* = 0.794, *P* = 0.373) (Table 3). The intake of dairy products in the frail group was greater than that in the nonfrail group (*Z* = 9.043, *P* = 0.003), and there was no statistically significant difference among the other groups (N vs. P, *Z* = 2.114, *P* = 0.146; P vs. F, *Z* = 2.919, *P* = 0.088). There were no significant differences in other food categories or main nutrients (*P* > 0.05) (Supplemental Table 1; supplemental material available online with this article; https://doi.org/10.1172/JCI176507DS1).

#### Differences in liver and kidney function and cytokine levels among groups with different frailty states.

Among the 16 biochemical indicators, 10 were significantly different: albumin, alanine aminotransferase (ALT), aspartate aminotransferase (AST), creatinine, cystatin C, direct bilirubin, globulin, indirect bilirubin, total bilirubin, and total protein. There were no significant differences in the levels of albumin/globulin, alkaline phosphatase, AST/ALT, urea, uric acid, or γ-glutamyltransferase among the 3 groups (*P* > 0.05). The level of albumin in the frail group was significantly lower than that in the nonfrail group (*P* = 0.003) or the prefrail group (*P* = 0.001). There were significant differences in creatinine among the 3 groups: compared with the nonfrail group, the prefrail group had an increase in creatinine (*P* = 0.027), whereas the frail group had a decrease in creatinine (*P* = 0.049), and the creatinine level of the frail group was significantly lower than that of the prefrail group (*P* < 0.001). For cystatin C, both the nonfrail group and the frail group had significantly lower cystatin C levels than did the prefrail group (*P* < 0.001). The serum globulin level was significantly lower in the frail group than in the nonfrail group (*P* = 0.006). However, total protein was significantly lower in the frail group than in the nonfrail and prefrail groups (*P* = 0.003 and *P* = 0.017, respectively). Direct bilirubin levels were significantly higher in the prefrail and frail groups than in the nonfrail group (*P* < 0.001 and *P* = 0.034, respectively). However, indirect bilirubin levels were significantly lower in the prefrail and frail groups than in the nonfrail group (*P* = 0.014 and *P* < 0.001, respectively). Total bilirubin levels were significantly lower in the frail group than in the nonfrail and prefrail groups (*P* < 0.001 and *P* = 0.011, respectively). ALT levels were significantly lower in the frail group than in the nonfrail and prefrail groups (*P* = 0.002 and *P* = 0.001, respectively). AST levels were also significantly lower in the frail group than in the nonfrail and prefrail groups (*P* < 0.001 and *P* = 0.002, respectively). In conclusion, from the nonfrail group to the frail group, albumin, globulin, total protein, ALT, AST, indirect bilirubin, and total bilirubin levels all gradually decreased with the progression of disease. However, creatinine, cystatin C, and direct bilirubin levels initially increased and then decreased.

In addition, there were significant differences in the levels of the 3 cytokines among the groups (*P* < 0.05). Compared with the nonfrail group, the prefrail group had significantly lower levels of IFN-γ (*P* = 0.005). The levels of IL-17 (*P* = 0.005), IFN-γ (*P* < 0.001), and insulin-like growth factor 1 (IGF-1) (*P* < 0.001) were significantly increased in the frail group. Compared with the prefrail group, IFN-γ levels in the frail group were significantly increased (*P* < 0.001) and IGF-1 levels were significantly decreased (*P* < 0.001). It is worth noting that in the process of disease progression from the nonfrail stage to the frail stage, IL-17 showed a gradual increase, whereas IFN-γ showed a trend of first decreasing and then increasing, and IGF-1 showed a trend of first increasing and then decreasing, which is worthy of further exploration (Table 4).

### Intestinal microbiota and metabolic characteristics in older individuals with different frailty states

Firmicutes, Bacteroidetes, Proteobacteria, and Actinobacteria were the main bacteria found among the older individuals (*n* = 300) (Table 5). As the degree of frailty changed, the species and abundance of the gut microbiota also changed. The relative abundance of *Bacteroides vulgatus*, *Ruminococcus bicirculans*, *Alistipes onderdonkii*, *Bacteroides ovatus*, *Bacteroides* *fragilis*, *Bacteroides caccae*, *Bacteroides thetaiotaomicron*, and *Bacteroides plebeius* increased substantially, while that of *Ruminococcus bromii*, *Lactobacillus ruminis*, *Anaerostipes hadrus*, *Eubacterium hallii*, and *Bifidobacterium adolescentis*, among others, decreased (Supplemental Figure 2).

The results of untargeted metabolomics detection of intestinal microbiota metabolites revealed a total of 664 metabolites, including 435 positive ion metabolites and 229 negative ion metabolites. There were 143 different metabolites between groups N and P, 50 between groups N and F, and 192 between groups P and F. The metabolite comparisons between groups are shown in Supplemental Figure 3 from both positive and anionic perspectives.

Kyoto Encyclopedia of Genes and Genomes (KEGG) analysis revealed that, compared with group N, metabolic pathways, including protein digestion and absorption, amino acid biosynthesis, ABC transport, mineral absorption, and alanine, tyrosine, and tryptophan biosynthesis were upregulated in group P (*P* < 0.05). Pyrimidine metabolism, pentose glucuronic acid conversion, and unsaturated fatty acid synthesis pathways were downregulated in group F (*P* < 0.05). Compared with group P, protein digestion and absorption, amino acid biosynthesis, ABC transport, and other differential metabolic pathways were downregulated in group F (*P* < 0.05) (Supplemental Figure 4).

### Comparison of baseline data between groups with different frailty states before intervention

There were no significant differences (*P* > 0.05) in age, sex, indicators of frailty, scales, or body composition between the placebo group and the probiotic mixture group in the prefrail group (*n* = 200) at baseline (Supplemental Table 2). Only oil intake was significantly different (*P* = 0.004), whereas the intake of other dietary components and major nutrients showed no statistical differences (*P* > 0.05) (Supplemental Table 3). There were statistically significant differences in the biochemical indices and cytokine levels of ALT, globulin, γ-glutamyltransferase, and IFN-γ (*P* < 0.05), but there were no significant differences in the other parameters (Supplemental Table 4).

No statistically significant differences (*P* > 0.05) were noted in terms of age, sex, the 5 indicators of frailty, scales, body composition tests, or the intake of major dietary and nutritional elements between the placebo group and the probiotic mixture group in the frail group at baseline (Supplemental Tables 5 and 6). Only cystatin C showed a significant difference (*P* = 0.006) in liver and kidney function and cytokine levels, whereas no significant difference was found in the other parameters (*P* > 0.05) (Supplemental Table 7).

Prebiotic intervention in older individuals with different frailty states (randomized, controlled trial)

After 12 weeks of intervention, the prebiotic mixture significantly improved frailty status in both the frail and prefrail groups, reduced exhaustion in the prefrail group, and improved walking speed in the frail group (*P* < 0.05). The body fat percentage significantly increased in the treatment arm, whereas muscle mass decreased in the placebo control arm among prefrail older individuals. Walking speed and grip strength improved in the treatment arm, while body fat increased and moisture percentage decreased in the placebo control arm among frail older people (*P* < 0.05) (Table 6). Urea and creatinine decreased, whereas globulin and total protein increased substantially in the treatment arm; γ-glutamyltransferase and indirect bilirubin increased in the placebo control arm among prefrail older individuals. Globulin levels increased, whereas IL-17 and IFN-γ levels showed a more obvious downward trend, although no statistically significant difference was found in the treatment arm; AST increased and cystatin C decreased in the placebo control arm among frail older individuals (Table 7). In summary, a prebiotic mixture can markedly increase protein levels in older individuals (both frail and prefrail), improve renal function (prefrail), and partially reduce inflammatory factors (frail).

The Table 8 lists the microbiota and metabolism after the interventions. There was no significant change in α-diversity among the groups after the intervention (*P* > 0.05). The results of the Tukey test showed that there was significant β-diversity between the subgroup in the prefrailty group that received maltodextrin placebo (PM group) after placebo intervention (PMA) and the subgroup in the prefrailty group that received prebiotic intervention (a prebiotic mixture of inulin and oligofructose) (PI group) after prebiotic intervention (PIA), as well as between the subgroup in the frailty group that received maltodextrin placebo intervention (FM group) after placebo intervention (FMA) and the frailty group that received prebiotic intervention (a prebiotic mixture of inulin and oligofructose) (FI group) after prebiotic intervention (FIA), regardless of the use of unweighted or weighted algorithms (*P* < 0.01) (Supplemental Figure 5). In the prefrail group, *B. adolescentis* was predominant in the prebiotic mixture group, and *Faecalibacterium* was predominant in the placebo group. In the PIA group, *Bifidobacterium pseudocatenulatum* and *E. coli*, *Veillonellaceae*, *Enterobacteriales*, and *Negativicutes* were the dominant bacteria (Supplemental Figure 6). There were 13 and 177 different metabolites between PMA and PIA and between FMA and FIA, respectively (*P* < 0.05) (Supplemental Figure 7). Compared with those in the FIA group, the overall expression of genes related to protein digestion and absorption, carbon metabolism in cancer centers, mineral absorption, aminoacyl tRNA biosynthesis, ABC transport, and amino acid biosynthesis in the FMA group tended to be downregulated (*P* < 0.05). A hierarchical clustering heatmap and network map showed that there were 25 different bacterial flora and 36 different metabolites between the PIA and PMA groups, as shown in Supplemental Figure 8, and the network diagram showed that *Dialister* was the key bacterial group in the core position and was markedly positively correlated with l-methionine, indole, l-alanine, l-tryptophan, phenyllactic acid, sorbitol and indole-3-lactic acid.

### Correlation analysis between intestinal flora and clinical indicators (randomized, controlled trial)

To further explore the correlation between the intestinal flora and clinical indicators, we screened the 30 most abundant operational taxonomic units (OTUs) and the expression data of the intestinal flora in the kingdom, phylum, class, order, family, genus, and species. The Pearson correlation algorithm was used to calculate the correlation between species and clinical indicators, and a heatmap was drawn. At the genus level, *Bifidobacterium* was positively correlated with grip strength, muscle mass, visceral fat, and albumin levels and negatively correlated with exhaustion and frailty scores. The abundance of *Blautia* in the family *Lachnospirillaceae* was positively correlated with grip strength and physical activity and negatively correlated with exhaustion and frailty scores and IL-17 levels. *Prevotella* was positively correlated with slow walking, exhaustion, and frailty scores, as well as with body water percentage, among others, and negatively correlated with grip strength, body fat percentage, and albumin levels, among others (Figure 1A). On the basis of the results of the aforementioned association analysis, we constructed a correlation network map. By examining the degree of connectivity depicted in the figure, we could discern core microorganisms and indicators. The findings revealed positive correlations between muscle mass and *Bifidobacterium*, *Ruminococcus*, and *Eubacterium halobium*. Grip strength was found to have positive associations with *Bifidobacterium*, *Peptostreptococcus*, *Ruminococcus*, and *Dorsiella*. IGF-1 was positively correlated with *Ruminococcus*, while albumin was negatively correlated with *Prevotella* and *Klebsiella* (Figure 1B). After intervention in the prefrail group, exhaustion was negatively correlated with *Ruminococcus* and positively correlated with *Enterococcus*. Weight loss was negatively correlated with *Veillonella*, and a slow walk was positively correlated with *Roseburia*, among others (Figure 1C). After intervention in the frail group, the PSQI was positively correlated with *Holdemanella*, whereas visceral fat, body fat percentage, and IFN-γ levels were positively correlated with *Fusobacterium* (Figure 1D).

### Correlation analysis between metabolites and clinical indicators (randomized, controlled trial)

**Figure 2:**
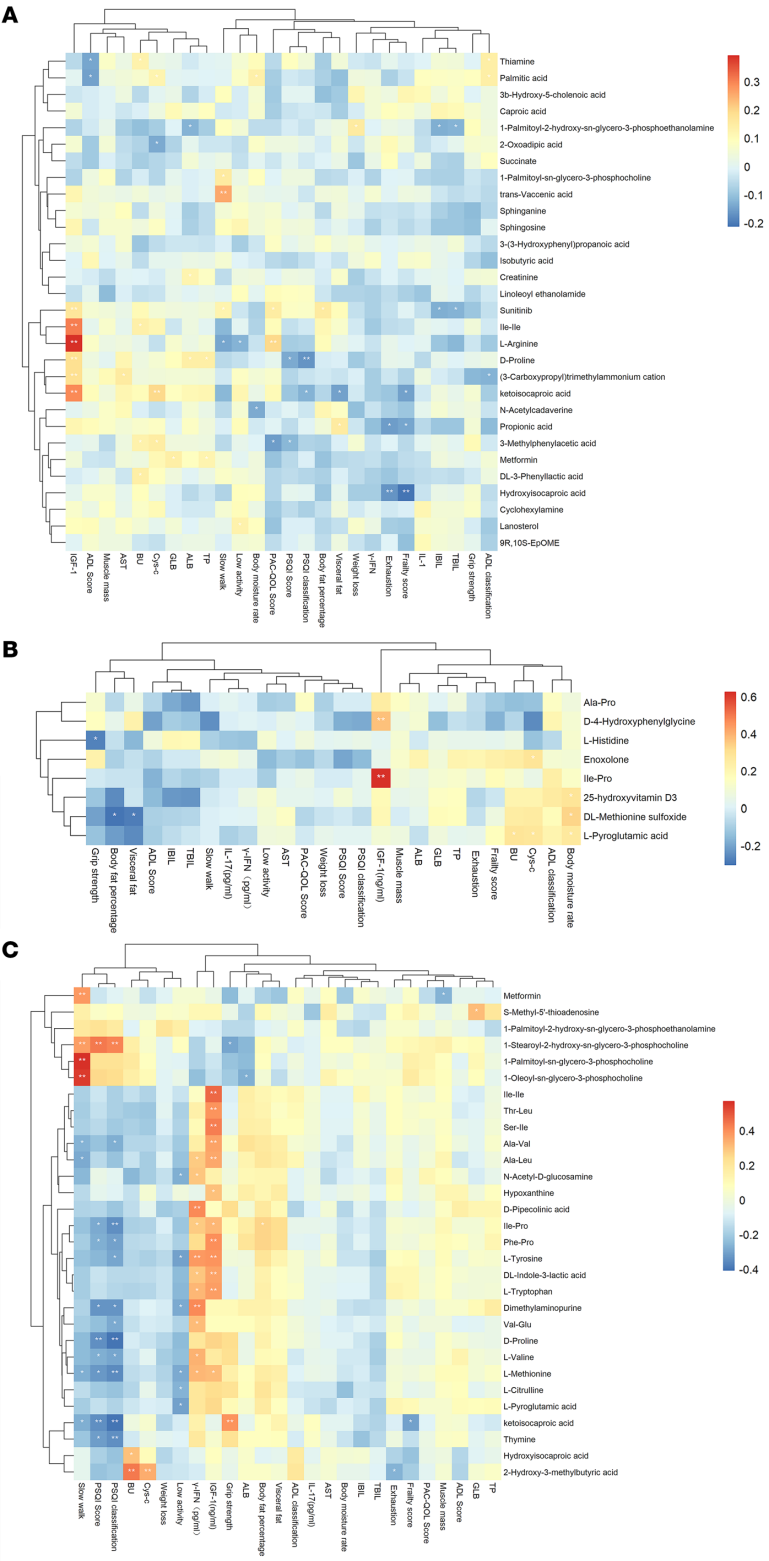
Correlation between the metabolites and clinical indicators. (**A**) Correlation between the most abundant metabolites (top 30) and clinical indicators. (**B**) Correlation between the metabolites and clinical indicators among prefrail older individuals (PIA vs. PMA). (**C**) Correlation between the metabolites and clinical indicators among frail older individuals (FIA vs. FMA).

Likewise, we screened the expression data of the top 30 most abundant metabolites and used the Pearson correlation algorithm to calculate the correlations between the metabolites and clinical indicators. The results showed that a slow walking speed was positively correlated with *trans*-vaccenic acid, the frailty score was negatively correlated with hydroxyisocaproic acid, and the PAC-QOL score was positively correlated with l-arginine (Figure 2A). The blood urea (BU) levels, cystatin C levels, and body moisture rate were positively correlated with l-pyroglutamic acid; body fat percentage, and visceral fat were negatively correlated with DL-methionine sulfoxide; and grip strength was negatively correlated with l-histidine among prefrail older individuals (Figure 2B). After intervention in the frail group, the core improvement in walking speed (walking time) was mainly positively correlated with metformin, 1-palmitoyl-*sn*-glycero-3-phosphocholine, and 1-oleoyl-*sn*-glycero-3-phosphocholine and was negatively correlated with l-methionine and ketoisocaproic acid (Figure 2C).

## Discussion

The intestinal microbiota — one of the most densely populated microbial communities on earth — contain highly diverse microbial communities that provide metabolic, immunologic, and protective functions that play a crucial role in human health. Increasingly, the consumption of prebiotics is recognized as an important avenue for modulating the composition and function of the intestinal microbiota (31). In this community-dwelling older adult–based clinical trial, we report an association between the intestinal microbiota and frailty status and further report that supplementation with prebiotics for 12 weeks resulted in a greater improvement in frailty status in older individuals than did placebo. The main mechanisms by which prebiotics may improve frailty status include modulation of the intestinal microbiota and metabolites.

We observed that advanced age, number of offspring, comorbidities, and history of surgery were negatively correlated with frailty severity and that exercise frequency was a protective factor. The intestinal microbiota of adults are dominated by Firmicutes and Bacteroidetes and smaller proportions of Proteobacteria, Actinobacteria, and Verrucomicrobia (32, 33). Previous studies have reported the enrichment of Bacteroidetes and Protobacteria and a decrease in Firmicutes and Bifidobacteria in older people. Rashidah et al. performed a systematic review in 2022 and showed that frail older individuals had lower intestinal microbiota diversity and a decreased abundance of Firmicutes, with *Dialister*, *Lactobacillus*, and *Ruminococcus* being the prominent genera, which is in accordance with our results (34, 35). We found that with increasing frailty severity, the relative abundance of Firmicutes decreased gradually, while that of Bacteroidetes increased. Via the capacity to generate either harmful or beneficial microbial metabolites, including short-chain fatty acids (SCFAs), cholate, and trimethylamine *N*-oxide (TMAO), as well as protein fermentation products, the intestinal microbiota have been linked to several diseases, such as inflammatory and cardiovascular diseases and metabolic disorders (33, 36–38). A general decrease in total energy, mass, and dietary protein intake metabolic pathways was found to be associated with frailty, as dietary protein intake and circulating amino acids are known to be components of muscle protein and are metabolic centers for several biological processes, including inflammation, insulin sensitivity, and redox homeostasis (39, 40). Our data also suggest a role for metabolic alterations as well as metabolic pathways in varying frailty states.

Given the potential plasticity of the intestinal microbiota, interventions involving diet, prebiotics, probiotics, and even fecal microbiota transplantation represent several promising directions. Ghosh et al. conducted a 12-month Mediterranean-style dietary intervention in 1,200 frail and nonfrail European participants from 65 to 79 years of age and found specific microbiome changes to be positively correlated with markers, such as reduced weakness and improved cognition, and negatively correlated with inflammatory markers (C-reactive protein [CRP], IL-17, etc.) (34). In our study, after a 12-week prebiotic intervention, we found that intervention using a prebiotic mixture reduced the exhaustion score and increased body fat and water percentages of older people in the prefrail group. In the frail group, walking speed and grip strength improved, as did symptoms of constipation. This indicates that a prebiotic mixture can improve the muscle strength of patients with frailty and has a certain effect on alleviating frailty status. In addition, no abdominal pain, diarrhea, or other symptoms were reported in the later period for any of the individuals who insisted on taking the prebiotic mixture, which also reflects the safety of the prebiotic mixture and the tolerance of its long-term use.

Our results showed that, in the prefrail group, the abundance of *B. adolescentis*, *Dorea longicatena*, *Eubacterium hallii*, *bacterium_LF-3*, *Lachnospiraceae_bacterium_1_1_57FAA*, and *Dorea formicigenerans* increased after prebiotic intervention. *B. adolescentis* is a probiotic bacterium that has recognized therapeutic effects on chronic diarrhea and constipation. It can also increase the activity and content of superoxide dismutase (SOD) in blood, reduce the damage of free radicals to human cells, and play an antiaging role (41, 42). The *Lachnospiraceae* family is known to participate in the breakdown of carbohydrates into SCFAs, which are believed to play a key role in microbiota-gut-brain cross talk and in the maintenance of intestinal barrier function. In addition, a clinical trial of fecal microbiota transplantation for ulcerative colitis in 2017 revealed that remission of symptoms was associated with enrichment of 2 bacteria: *Eubacterium hallii* and *Roseburia inulinivorans*. These microorganisms are thought to promote SFCA production and starch breakdown (43). Interestingly, LefSe analysis showed that in the frail group, prebiotics increased the abundance of the probiotic *Bifidobacterium pseudosmall chain*; moreover, the abundance of *E. coli*, a bacterium commonly considered to be an opportunistic pathogen, was also relatively increased. Kong et al. characterized the microbiota of a group of long-lived people (90 years of age or older) in Dujiangyan, China, and revealed that the long-lived group had a greater diversity of gut microbiota than did the young adult group. Further in-depth characterization of the microbiota in the long-lived cohort revealed that several known SCFA-producing bacteria were enriched. However, this result was accompanied by a decrease in some commonly beneficial bacteria (e.g., *Faecalis*) and an increase in some potentially pathogenic bacteria (e.g., *E. coli* and *Shigella*) (44). This finding is consistent with our findings, suggesting that the synergistic or antagonistic effects of the microbiota are complex and that maintaining a diverse and balanced gut microecology may be a key factor in ameliorating frailty (44). We further assessed target metabolism, which showed that prebiotic intervention increased the levels of methionine, histidine, and alanine and that the changes in metabolites tended to be similar to those in nonfrail or nonsarcopenic individuals. *Dialister* is key within the microbiota and is significantly positively correlated with indole, l-alanine, l-tryptophan and indole-3-lactic acid.

Although our study had several strengths, including a randomized trial design, double-blinded intervention, and rigorous assessment, there were several limitations. There is no clear gold standard for frailty assessment at present; thus, the Chinese version of the Fried Frailty Assessment Method recommended by the Chinese Expert Consensus on Frailty Assessment and Intervention for Elderly Patients was used in our study. It is plausible that the screening method is unsuitable for bedridden older individuals with obvious body dysfunction, and that the use of the bioimpedance analysis for body composition is a less precise measure than CT or MRI. Due to the possible delay between microbiome and metabolome changes, association studies between the metabolome, microbiome, and host state must be performed with caution, and clinical studies aimed at the intervention of metabolites are lacking. Further microbiota studies should include the influence of other phyla (such as viruses, fungi, and archaea), as well as other host characteristics (such as ethnic origin, nutrition, and genetics).

### Conclusions.

This clinical trial showed that the diversity, composition, and function of the intestinal microbiome varied with frailty status in community-dwelling older adults in China. Intestinal microbiome dysbiosis is linked to frailty status, and prebiotic mixture interventions may be a promising direction for treatment. Future studies are needed to determine whether other microorganisms participate in the frailty process and which combined therapy with prebiotics may further improve outcomes.

## Methods

### Sex as a biological variable.

Our research included both men and women.

### Design.

**Figure 3:**
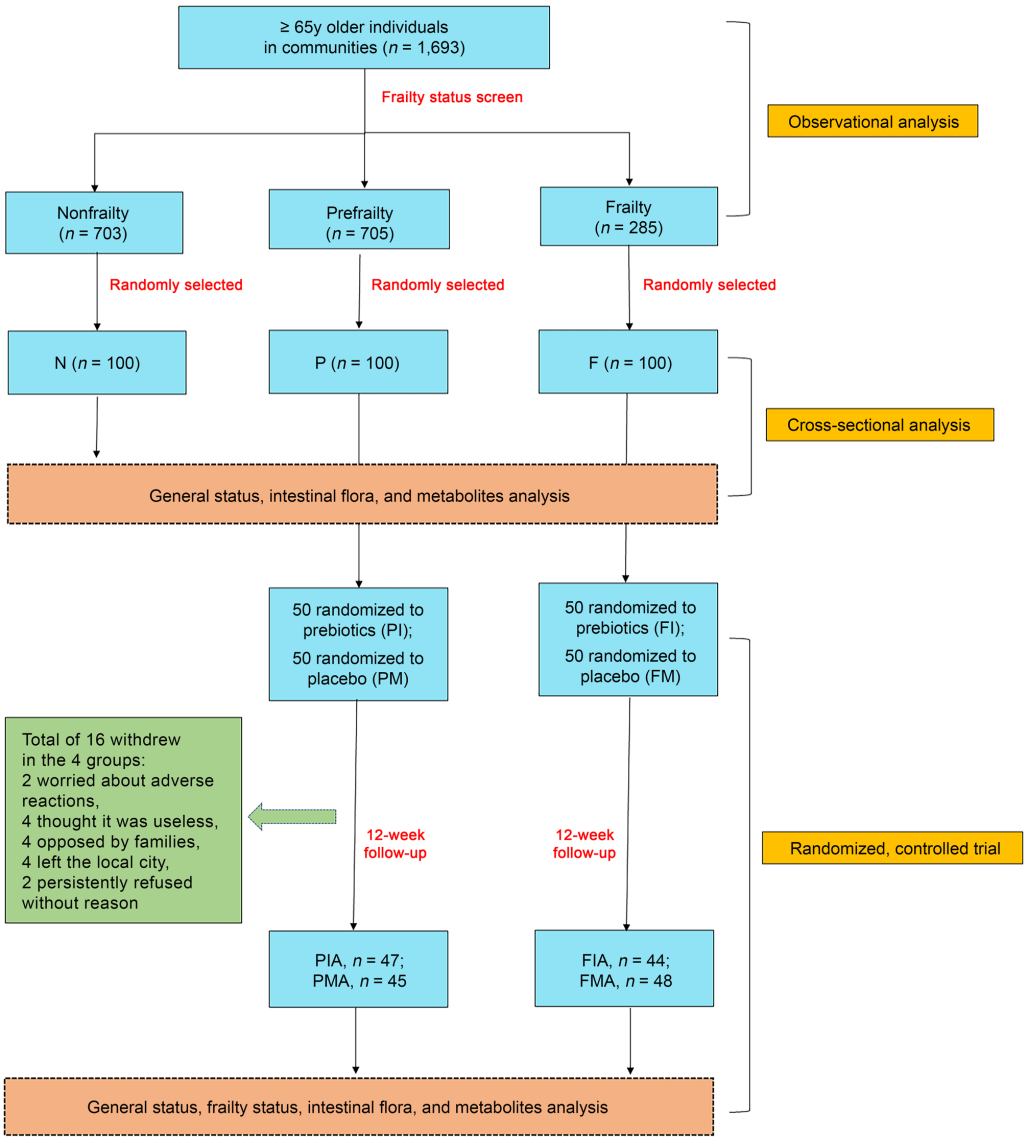
Trial profile.

We conducted a large multicenter, double-blind, randomized, placebo-controlled trial involving community-dwelling older individuals. The study, conducted at 13 communities in Xi’an, China, was mainly divided into 3 parts: an observational analysis involving 1,693 participants, a cross-sectional analysis with 300 participants, and a randomized, controlled trial comprising 200 participants, which was prespecified in the trial protocol (Figure 3).

### Participants.

The study was conducted in the 13 communities of Xi’an city, Shaanxi Province, China. Community-dwelling older individuals who were over 65 years of age were recruited and clinically followed between August 11, 2018, and December 30, 2020. All individuals agreed to participate in the survey. The inclusion criteria were as follows: (a) able to get up from a chair and walk at least 6 meters with or without a walking device; (b) had normal cognitive ability, with the ability to read and express themselves (Researchers had brief conversations with the participants, including asking for their name, address, family information, current season, etc. Second, the prospective participant read a paragraph of text from a newspaper aloud. Those who could read it correctly were considered qualified.); and (c) agreed with the research program and were willing to participate in the survey. The exclusion criteria were as follows: (a) blindness, (b) acute infection, (c) cancer or other serious disease (if the cancer had responded well after surgery, no distant metastasis was found, and no chemotherapy, radiotherapy, or special drugs were needed, the individual was not excluded), or (d) dementia, severe cognitive dysfunction, or mental illness, and were unable to live on their own or were unwilling to participate in the survey.

### Randomization and interventions.

PASS11 software was used to calculate sample size, with β = 0.2 and α = 0.05. Fifty participants were planned to be recruited in each group (*n* = 50 participants in the intervention group and *n* = 50 participants in the placebo group), with a total of 100 participants to be recruited in the prefrail group and 100 to the frail group. Additional exclusion criteria were as follows: acute or chronic inflammatory disease of the intestines in the previous 3 months; use of antibiotics, probiotics, prebiotics, or synbiotics in the past month; use of laxatives or drugs for diarrhea, proton pump inhibitors, or gastric motility drugs for more than 3 days. Randomization was performed using a dynamic allocation method; random numbers were stratified by sex at a 1:1 ratio, and the details of the allocation sequence and the allocation group were concealed from the researchers. Independent study coordinators dispensed either placebo or prebiotics according to a computer-generated, randomized sequence. The computer automatically generated a random number for each of the 100 persons in the group and then ranked the numbers in descending order, with the first 50 individuals taking the intervention mixture (a prebiotic mixture of inulin and oligofructose [50% each] derived from chicory, 15 g/d, taken after breakfast) and the last 50 individuals taking the placebo (maltodextrin, 15 g/d, taken after breakfast). All participants, study coordinators, and researchers were blinded to the intervention arm throughout the entire study.

### Outcomes.

The primary outcomes included the results from the frailty scale. The secondary outcomes were the gut microbial composition and metabolite pathways at the beginning and end of the experiment. Information on the participants’ exercise, diet, and other living habits and general conditions was obtained via a questionnaire, and the investigators received unified training before conducting the survey. Body fat percentage, muscle mass, and visceral fat were measured by a body composition analyzer.

### Screen of frailty status.

The Fried scale was used to define frailty status (45), which included (a) unintentional weight loss (unintentional weight loss >4.5 kg or 5% of body weight in the past year); (b) exhaustion (evaluated using the Center for Epidemiological Survey, Depression Scale [CES-D]). That is, the score for any 1 of the 2 questions in CES-D was 2–3 points, which could be determined as the score for the item: (a) I feel like I need to work hard in everything I do; (b) I cannot walk forward (0 score: <1 day; 1 point: 1–2 days; 2 points: 3–4 days; 3 points: >4 days); (c) decreased grip strength (determined by sex and BMI, judged on the basis of the average of the 3 grip strength levels of the dominant hand. Men: BMI ≤24.0 kg/m^2^, weight ≤29 kg; BMI 24.1–28.0 kg/m^2^, weight ≤30 kg; BMI >28 kg/m^2^, weight ≤32 kg. Women: BMI ≤23.0 kg/m^2^, weight ≤17 kg; BMI 23.1–26.0 kg/m^2^, weight ≤17.3 kg; BMI 26.1–29.0 kg/m^2^, weight ≤18 kg; BMI >29 kg/m^2^, weight ≤21 kg); (d) decreased walking speed (time it took to walk 4.57 meters, by sex and height. Men: height ≤173 cm, ≥7 seconds; height >173 cm, ≥6 seconds; women: height ≤159 cm, ≥7 seconds; height >159 cm, ≥6 seconds); and (e) reduced physical activity (assessed according to the Minnesota Leisure Time Physical Activity (MLTA) questionnaire by sex. Men: <383 kcal/week [~2.5 hours of walking]; women: <270 kcal/week [~2 hours of walking]). Specifically, the grip strength was measured by a Jamar Plus+ grip dynamometer, and all of these indicators were assessed by face-to-face surveys. The presence of 1–2 indicators in older individuals was defined as prefrail, and more than 3 indicators was defined as frail.

### Sociodemography and general health status, geriatric scale, “bioimpedance analysis” of body composition variables, daily diet survey, hepatic and renal function, and cytokines.

The sociodemographic and general health status of the participants was assessed in person by staff and included name, sex, date of birth, phone number, ethnicity, occupation, education level, marital status, number of children, and frequency of social visits to the older individual by children, relatives, and friends. The frequency of visits was categorized as follows: (a) regular: at least once weekly; (b) occasional: once or twice monthly; (c) infrequent: once or twice annually; (d) seldom: not visited for over a year. Visits falling under the first category were deemed “frequent,” whereas the remaining 3 categories were amalgamated as “other”. Other information collected included per capita monthly income of the family and living situation, which was categorized as follows: (a) living alone; (b) living with spouse; (c) living with children; (d) living with both spouse and children; (e) other situation (participant was asked to specify). These living situations were further divided into 2 groups: (a) living alone and (b) living with others. The remaining categories were as follows: smoking status, alcohol consumption, exercise frequency, personality type, surgical history, medical history, medication status, and occurrence of acute events.

The common scales for older persons used in this study included the Activities of Daily Living (ADL) (46), the Self-rating Anxiety Scale (SAS) (47), the Geriatric Depression Scale (GDS) (48), Patient Assessment of Constipation–Quality of Life (PAC-QOL) scale (49), and the Pittsburgh Sleep Quality Index (PSQI) (50). The above scales are all internationally recognized and rated according to the corresponding standards for judgment. The detection by bioimpedance analysis (BIA) of body composition variables was strictly controlled by the staff on the basis of the indications. During detection, the older person was allowed to stand on a body composition analyzer (Tanita BC-545N) according to the instructions. The stabilization of the test reading allowed for the sequential recording of various indices, including weight (kg), BMI (kg/m^2^), fat percentage, muscle mass (kg), bone mass (kg), visceral fat grade, basal metabolism (kcal), and body moisture percentage.

The daily diet survey was conducted using the “3-Day 24-hour Diet Record Questionnaire,” which recorded all the food eaten on Thursday, Friday, and Saturday for 3 consecutive days and entered on a separate sheet for each day. The researchers explained the recording method to the study participants on site and combined it with the food model to help the participants establish the concept of the specific weight of food, emphasizing that all food eaten at home or outside of meals (including snacks) should be entered in the survey table to avoid omissions. After confirming that the individual was able to accurately understand the form, the form was handed to the person or their caregiver. Finally, the outpatient nutrition guidance software program (version 3.0), developed by Shanghai Zhending Health Technology Co., was used to input and export the data.

Biochemical indicators and cytokines were detected in the participants’ blood samples. Fasting venous blood was drawn from all participants in the morning and centrifuged immediately thereafter. The serum isolated from each sample was divided into 2 parts, with 1 part immediately sent for biochemical function detection to the clinical laboratory at Xijing Hospital, which is affiliated with the Air Force Military Medical University; the other part was used for double-antibody sandwich ELISA (Wuhan Elabscience Biotechnology) to detect cytokines.

### Determination of fecal microbiota.

Fecal samples were collected at baseline and at the end of the 3-month intervention. The detailed fecal collection process and matters needing attention were formulated, and the researchers and participants were trained at the beginning of the experiment. Each fecal sample (approximately 2 g) was collected by participants at home in a dry, aseptic exclusive stool collector, immediately stored at –20°C, and sent within 1 hour through the cold chain (–80°C) until analysis. DNA was extracted from fecal samples using the SDS method and detected by 16S rDNA gene sequencing. PICRUSt software was used to analyze the function of the gut microbiota. High-throughput chromatography‒mass spectrometry was used to assess the metabolomics of the targeted microbiota.

### Statistics.

Descriptive statistics, including the mean, median, and ranges, were used to describe all the quantitative variables. Categorical variables are described by proportions; medians and interquartile distances were used to describe continuous variables that were not normally distributed. ANOVA was used to test the differences between groups of continuous variables, the χ^2^ test was used to test the differences between groups of categorical variables, and the rank-sum test was used to test non-normally distributed data. Statistical significance was set at a *P* value of less than 0.05, and statistical analysis was performed using SPSS 25.0 software.

### Study approval.

This study conformed to the ethics principles stated in the Declaration of Helsinki, and the project was approved by the Ethics Committee of the First Affiliated Hospital of Air Force Medical University (KY20192015-F-1) and registered in the US Clinical Trial Registry (NCT03995342). All participants were informed of and consented to the study protocol.

### Data availability.

The primary data that substantiate the findings presented in this work can be found in the Supporting Data Values file. The data supporting the findings of this study are available from the corresponding author upon reasonable request.

## Author contributions

JY and XW conceptualized and designed the study. AW, JY, LH, LS, XJ, RX, and XW acquired and analyzed the data. JY and LH wrote the manuscript. AW, JY, LH, LS, XJ, RX, and XW critically revised the manuscript and approved the final version of the manuscript. XW agreed to be accountable for all aspects of the work and ensured that questions related to the accuracy or integrity of any part of the work were appropriately investigated and resolved.

## Supplementary Material

Supplemental data

ICMJE disclosure forms

Supporting data values

## Figures and Tables

**Figure 1:** Correlation between the flora and clinical indicators. (**A**) Correlation between the most abundant flora (top 30) and clinical indicators. (**B**) Correlation network map between the flora (top 30) and clinical indicators. This network diagram is based on Pearson correlation analysis to calculate the correlation between clinical indicators and intestinal flora and is drawn with a *P* value of less than 0.5. The red circle represents the microorganism, the blue box represents the clinical index, and the size of nodes represents the size of the surrounding connectivity. The more connections, the larger the size. The solid line represents positive correlation, whereas the dashed line represents negative. (**C**) Correlation between the flora and clinical indicators among prefrail older individuals (PIA vs. PMA). (**D**) Correlation between the flora and clinical indicators among frail older individuals (FIA vs. FMA).
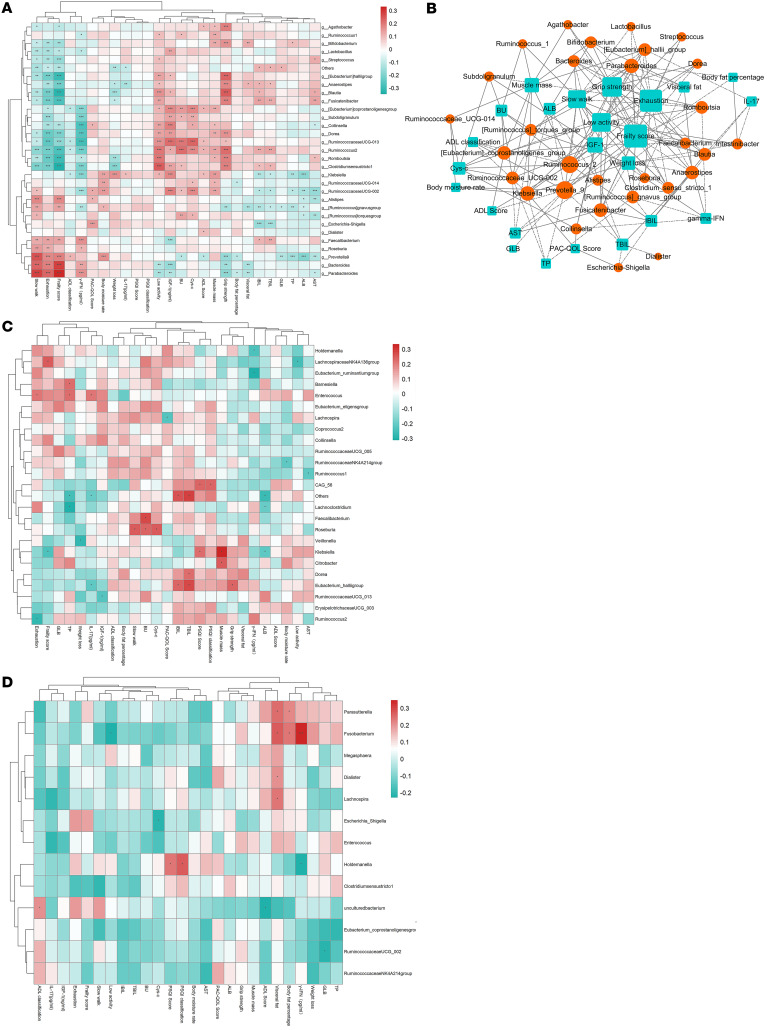

**Table 8:**
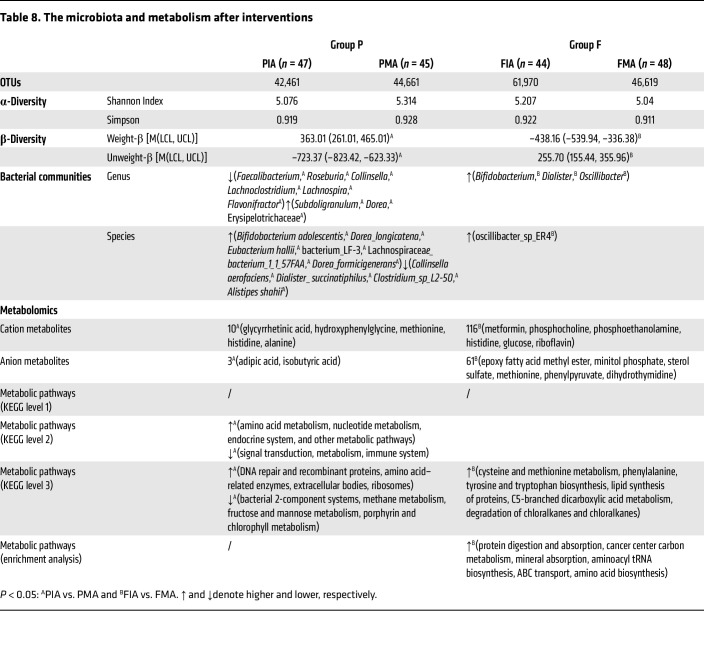
The microbiota and metabolism after interventions

**Table 7:**
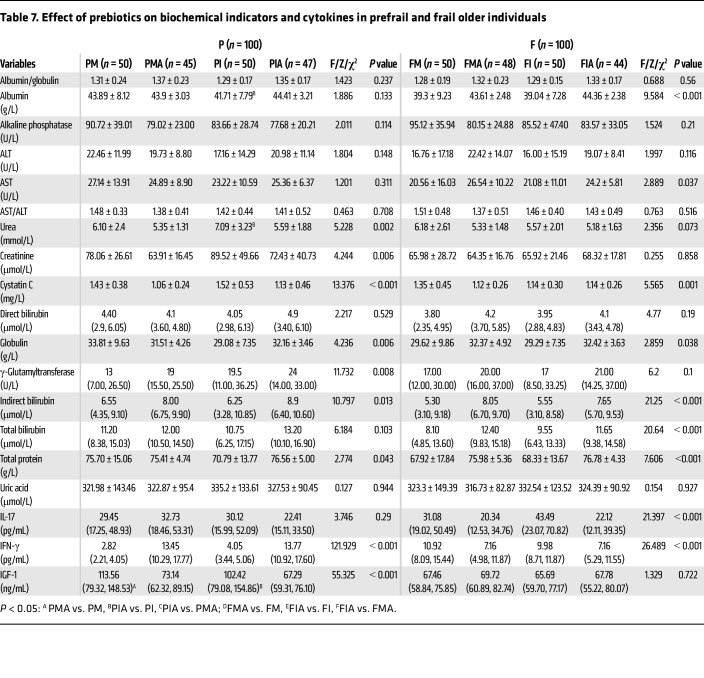
Effect of prebiotics on biochemical indicators and cytokines in prefrail and frail older individuals

**Table 6:**
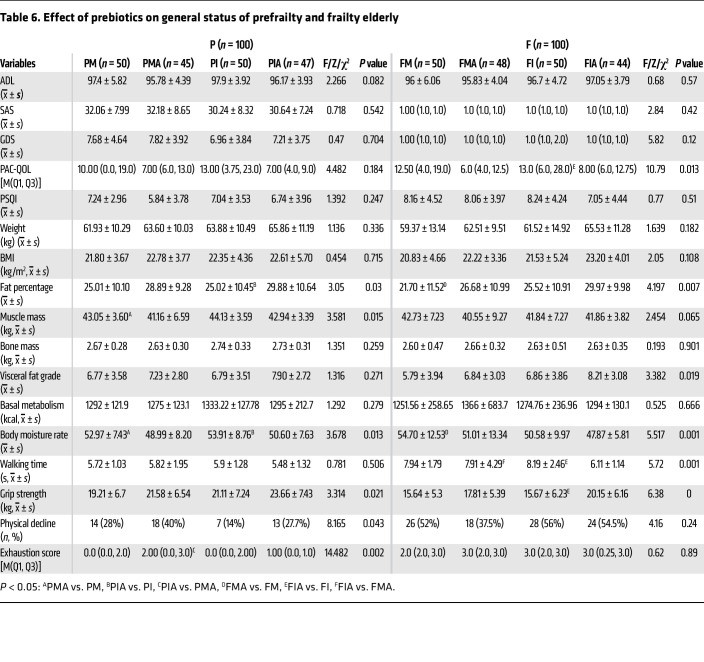
Effect of prebiotics on general status of prefrailty and frailty elderly

**Table 5:**
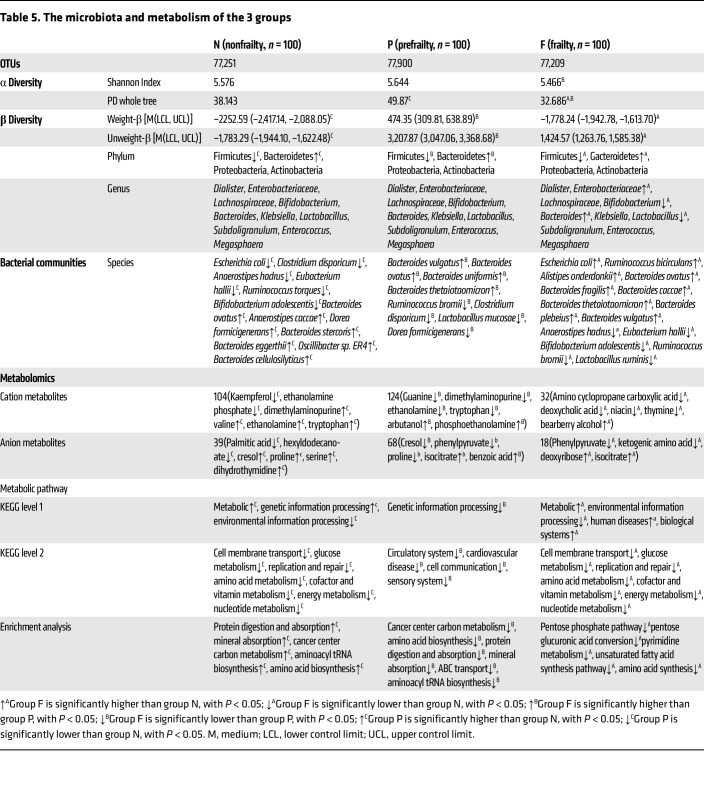
The microbiota and metabolism of the 3 groups

**Table 4:**
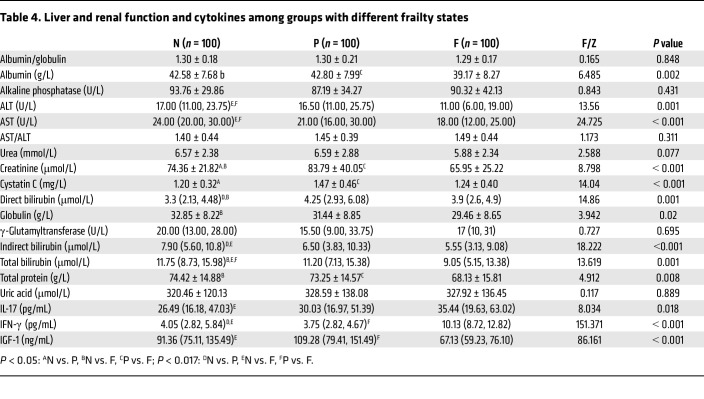
Liver and renal function and cytokines among groups with different frailty states

**Table 3:**
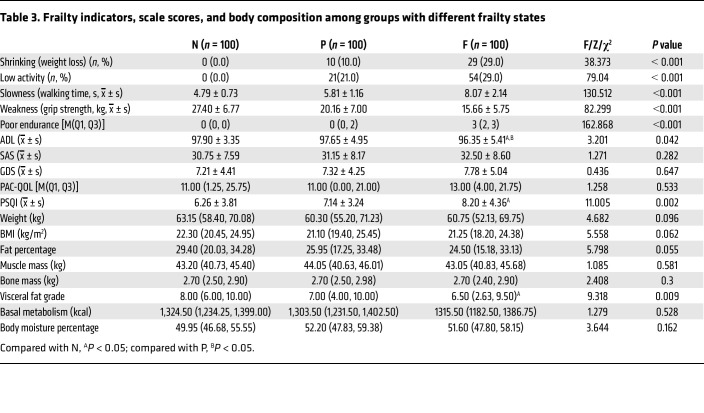
Frailty indicators, scale scores, and body composition among groups with different frailty states

**Table 1:**
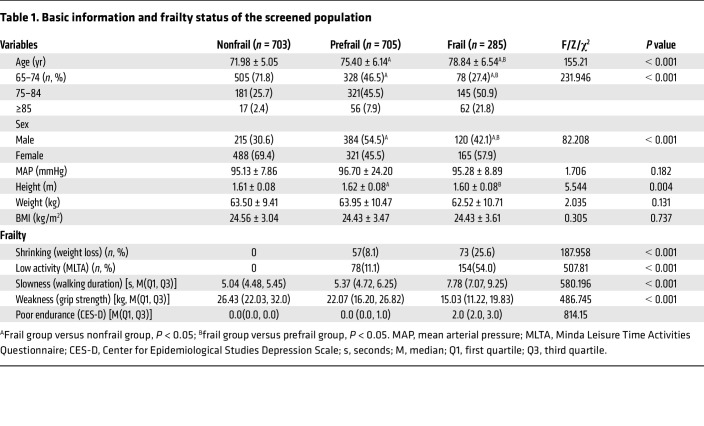
Basic information and frailty status of the screened population

**Table 2:**
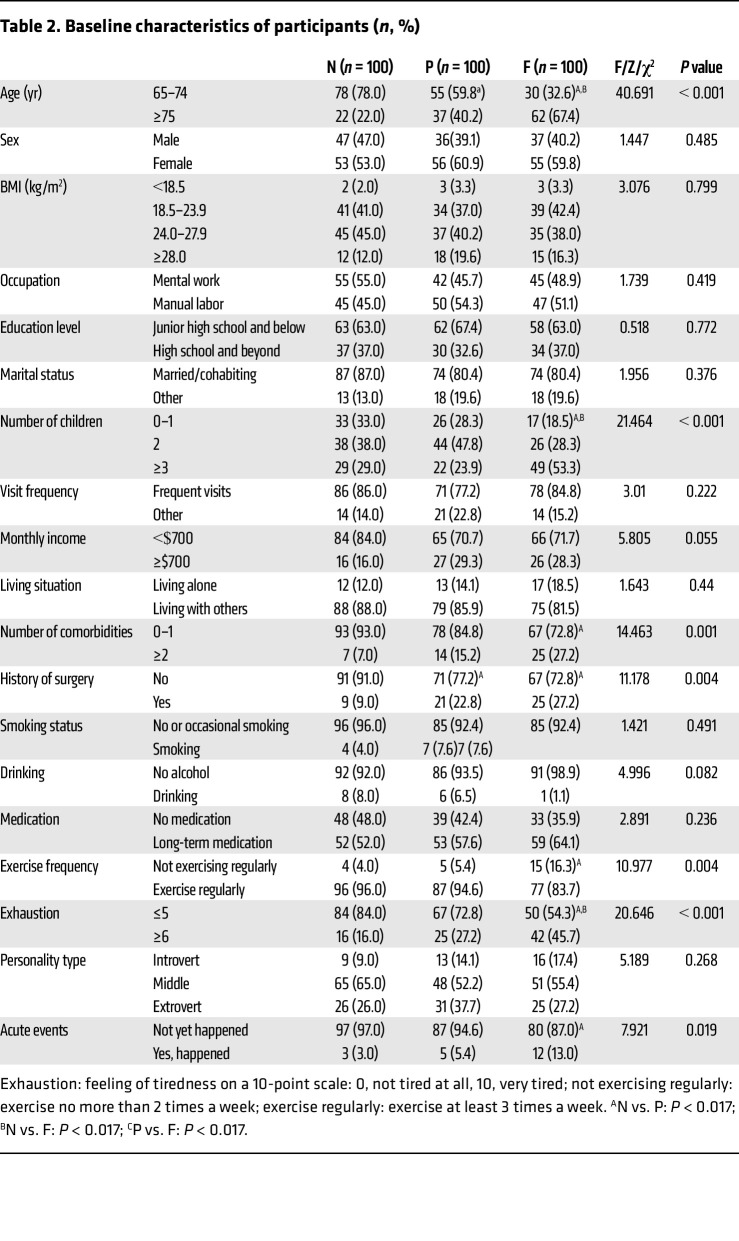
Baseline characteristics of participants (*n*, %)

## Version 1 09/17/2024

Electronic publication

## Version 2 09/18/2024

Typographical error corrected in affiliations list.
