# Supplementary material for: Prebiotics improve frailty status in community-dwelling older individuals in a double-blind, randomized, controlled trial
Source: J Clin Invest. 2024 Aug 6;134(18):e176507. doi: 10.1172/JCI176507 (PMC11405044; doi:10.1172/JCI176507)
Supplement: Supplemental data [file jci-134-176507-s012.pdf]

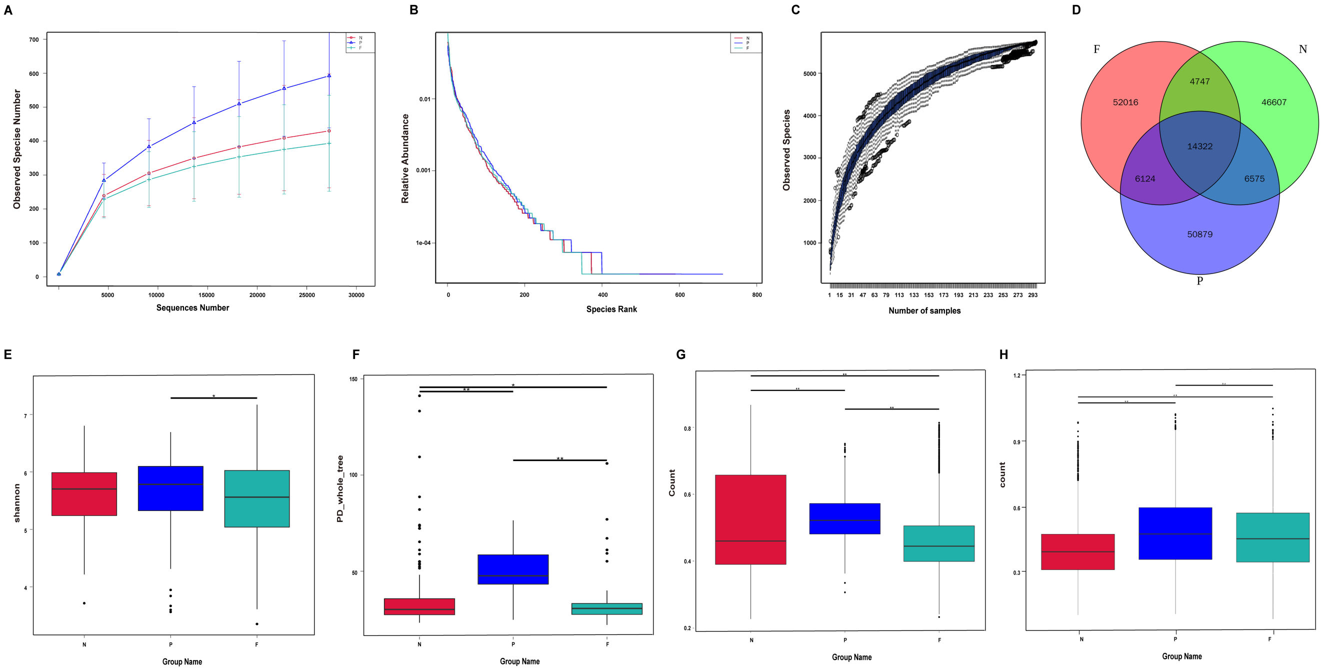

Supplemental Figure1 OTU analysis and diversity between three groups

N: nonfrailty, P: prefrailty, F: frailty; A: Block dilution curve, B: Group rank clustering curve, C: Species accumulation box chart, D: Venn diagram of OTUs distribution in three groups, E: Shannon index box plot of differences between groups, F: Box plot of differences between groups in PD Whole Tree index, G: Group differences in unweighted Beta diversity, H: Group differences in weighted Beta diversity

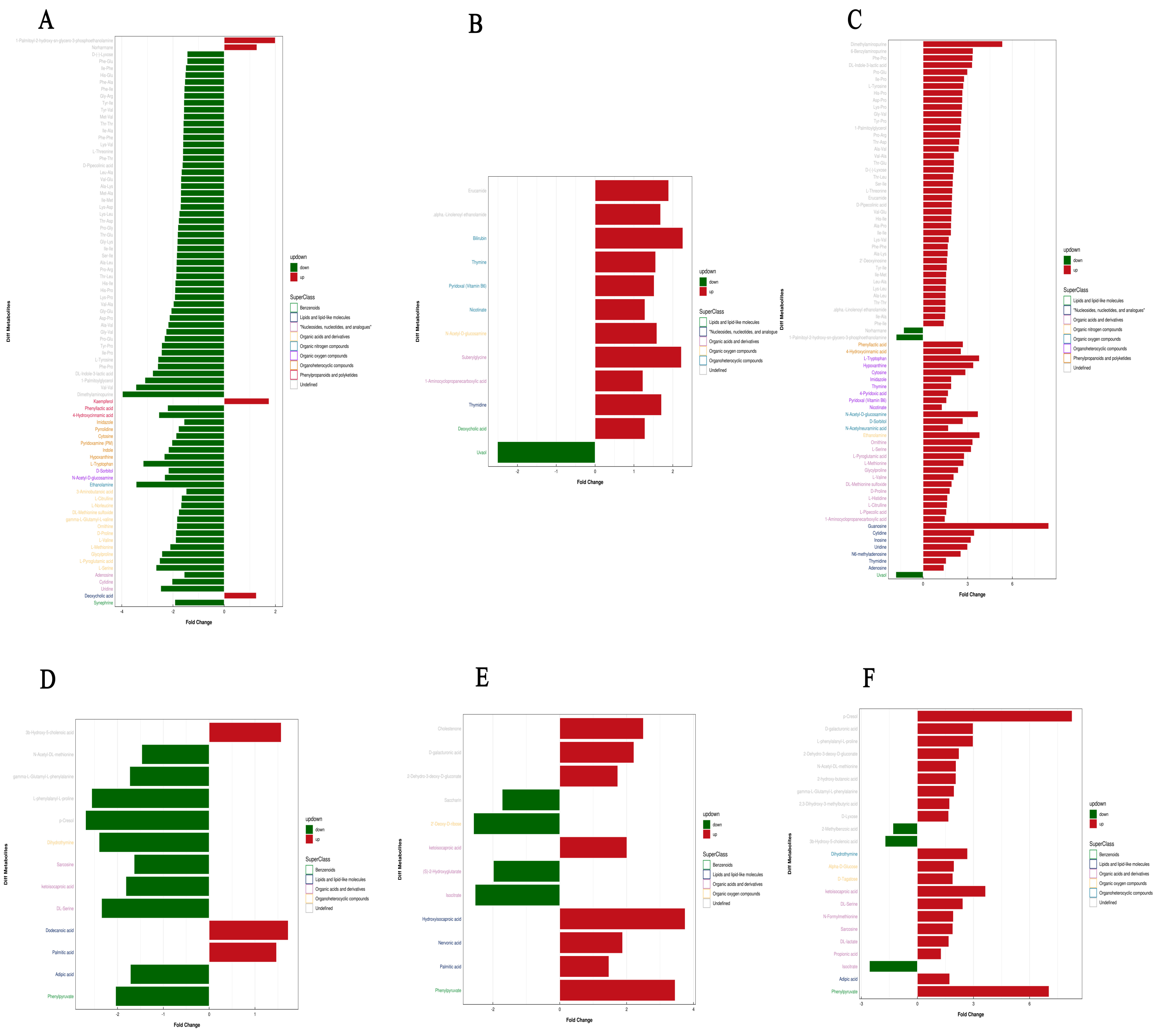

Supplemental Figure2 The abundance of bacteria and species analysis between the three groups

N: nonfrailty，P: prefrailty，F: frailty；A: Bar chart of relative abundance of dominant species pairs from Phylum level, B: Bar chart of relative abundance of dominant species pairs from Genus's level, C: Bar chart of relative abundance of dominant species pairs from Species level, D: Genus level microbiota clustering heat map, E: Family level microbiota clustering heat map, F: Phylum level analysis between group N and P, G: Phylum level analysis between group N and F, H: Phylum level analysis between group P and F, I: Species level analysis between group N and P, J: Species level analysis between group N and F, K: Species level analysis between group P and F, L: Evolutionary branching diagram of groups N and F, M: Evolutionary branching diagram of groups P and F

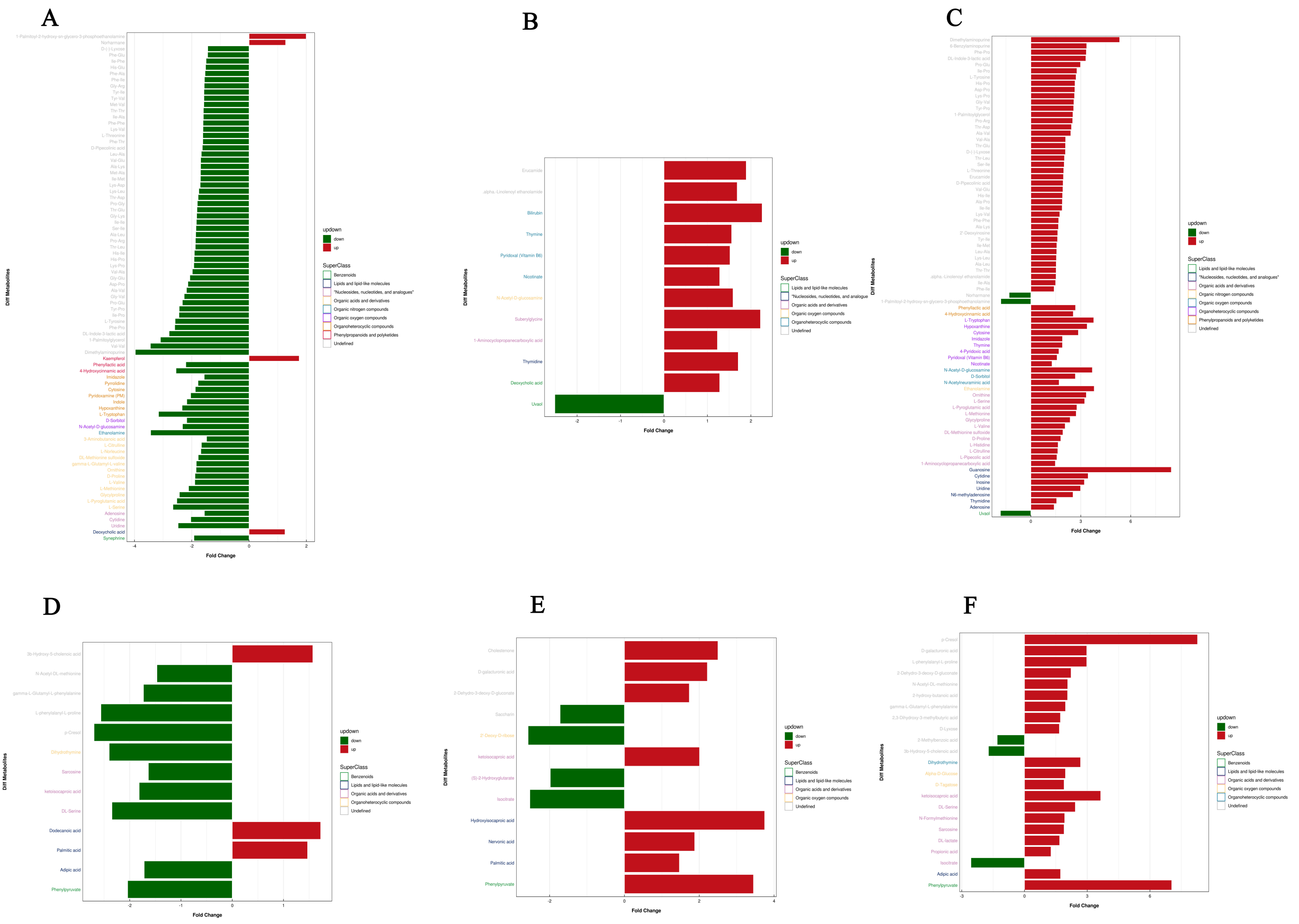

Supplemental Figure3 The metabolites between groups of different frailty states

N: nonfrailty，P: prefrailty，F: frailty；A: Positive ion mode between group N and P, B: Positive ion mode between group N and F, C: Positive ion mode between group P and F, D: Anion ion mode between group N and P, E: Anion ion mode between group N and F, F: Anion ion mode between group P and F)

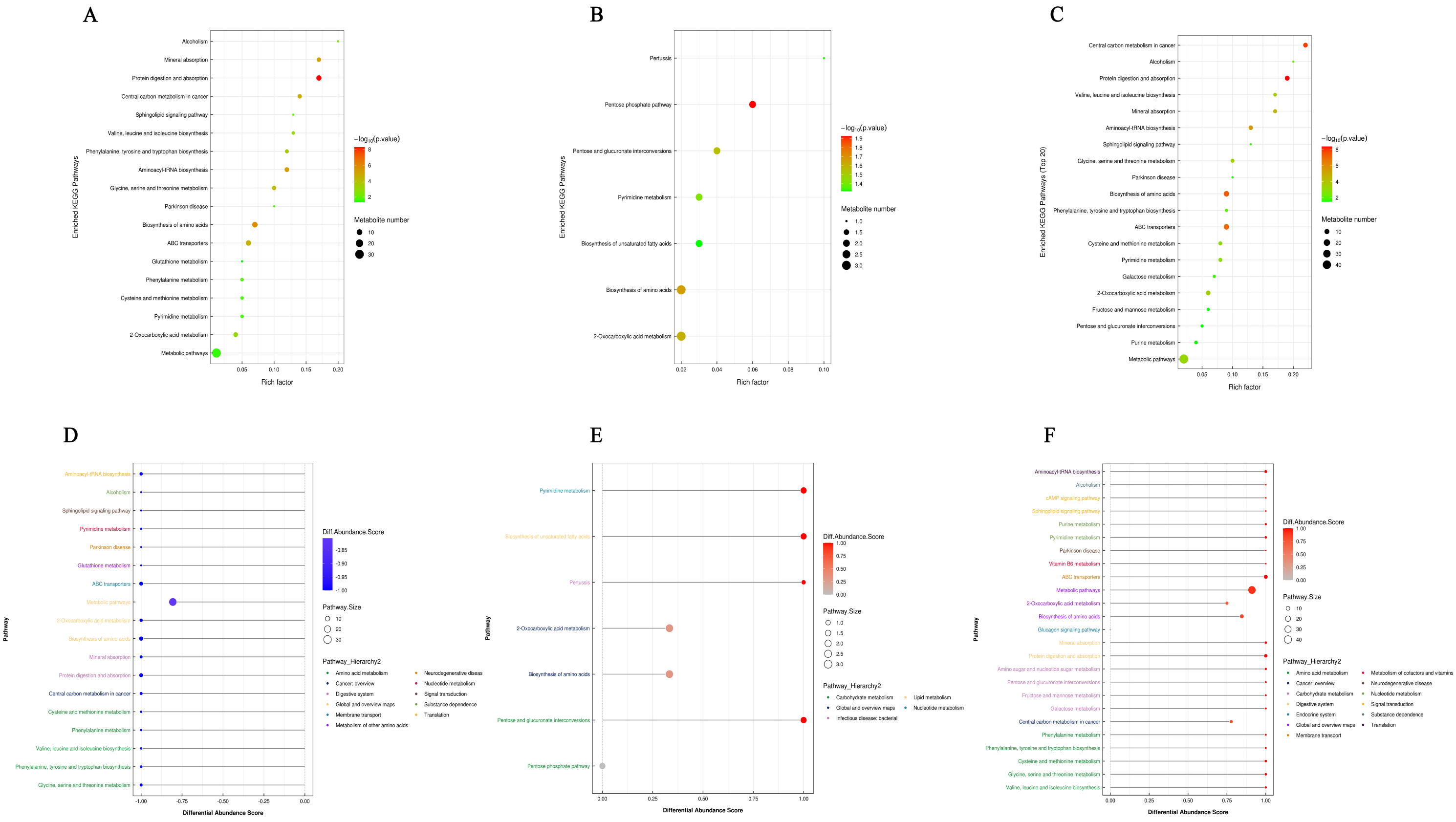

Supplemental Figure 4 KEGG pathway enrichment analysis

N: nonfrailty, P: prefrailty, F: frailty; A: Bubble diagram of KEGG enrichment pathway between group N and P, B: Bubble diagram of KEGG enrichment pathway between group N and F, C: Bubble diagram of KEGG enrichment pathway between group P and F, D: Differential abundance score plot of all differential metabolic pathways between group N and P, E: Differential abundance score plot of all differential metabolic pathways between group N and F, F: Differential abundance score plot of all differential metabolic pathways between group P and F

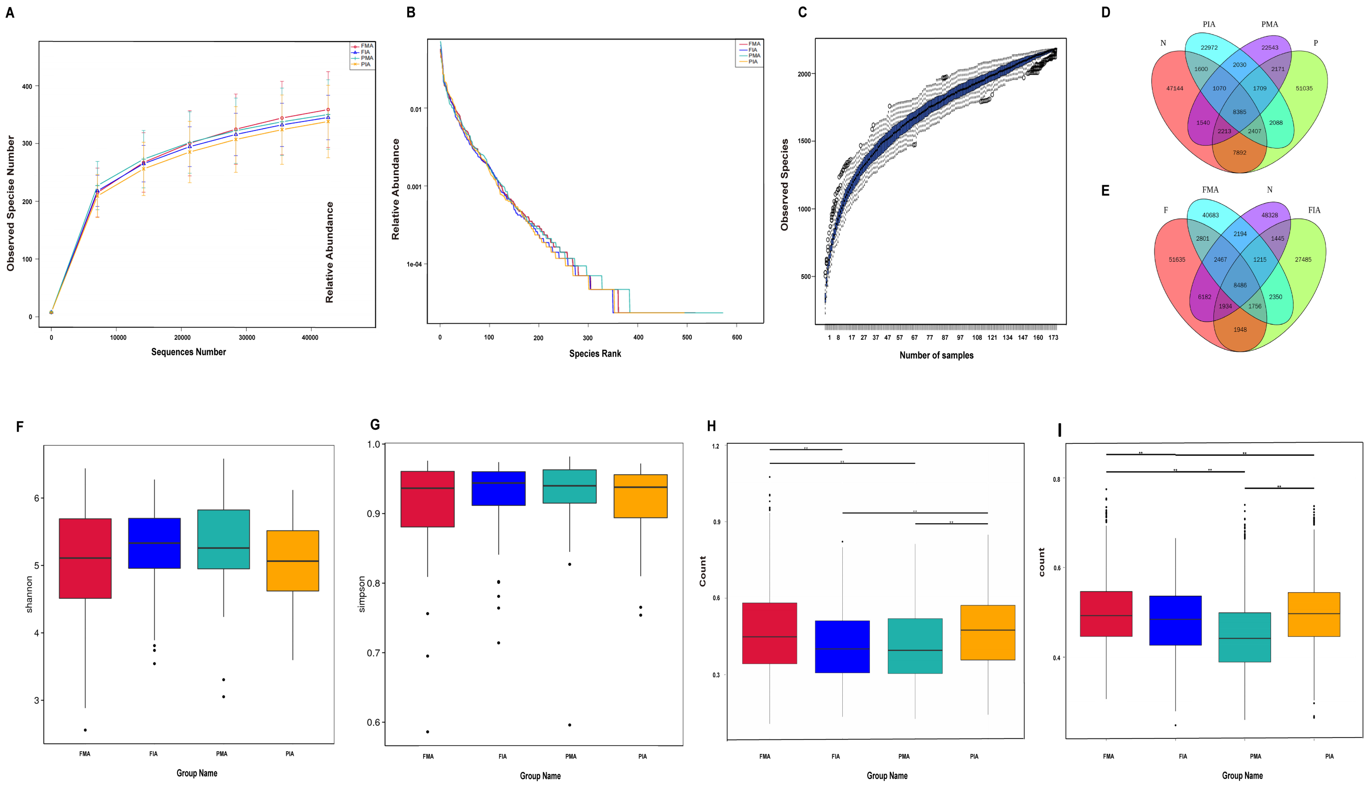

Supplemental Figure 5 OTU analysis and diversity between four groups after interventions

PI: prefrailty group before receive prebiotic intervention; PM: prefrailty group before receive placebo intervention; FI: frailty group before receive prebiotic intervention; FM: frailty group before receive placebo intervention; PIA, PI group after receive prebiotic intervention. PMA, PM group after receive placebo intervention. FIA, FI group after receive prebiotic intervention. FMA, FM group after receive placebo intervention. A: Block dilution curve, B: Group rank clustering curve, C: Species accumulation box chart, D and E: Venn diagram of OTUs distribution between groups, F: Shannon index box plot of differences between groups, G: Simpson index box plot of differences between groups, H: Group differences in weighted Beta diversity, I: Group differences in unweighted Beta diversity

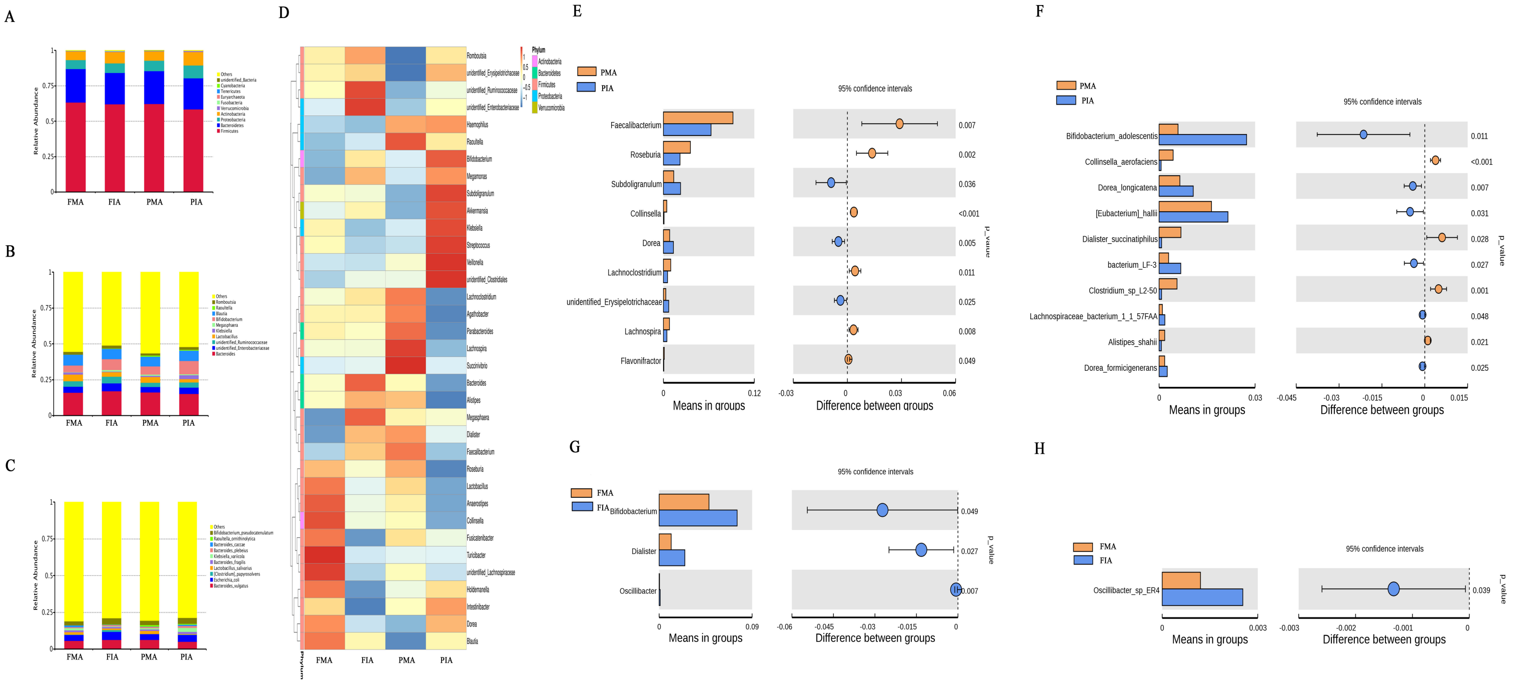

Supplemental Figure 6 The abundance and species analysis of bacteria in four groups after interventions

PI: prefrailty group before receive prebiotic intervention; PM: prefrailty group before receive placebo intervention; FI: frailty group before receive prebiotic intervention; FM: frailty group before receive placebo intervention; PIA, PI group after receive prebiotic intervention. PMA, PM group after receive placebo intervention. FIA, FI group after receive prebiotic intervention. FMA, FM group after receive placebo intervention. A: Bar chart of relative abundance of dominant species pairs (Phylum level), B: Bar chart of relative abundance of dominant species pairs (Genus level), C: Bar chart of relative abundance of dominant species pairs (Species level), D: Genus level microbiota clustering heat map, E: Genus level analysis between group PMA and PIA, F: Species level analysis between group PMA and PIA, G: Genus level analysis between group FMA and FIA, H: Species level analysis between group FMA and FIA.

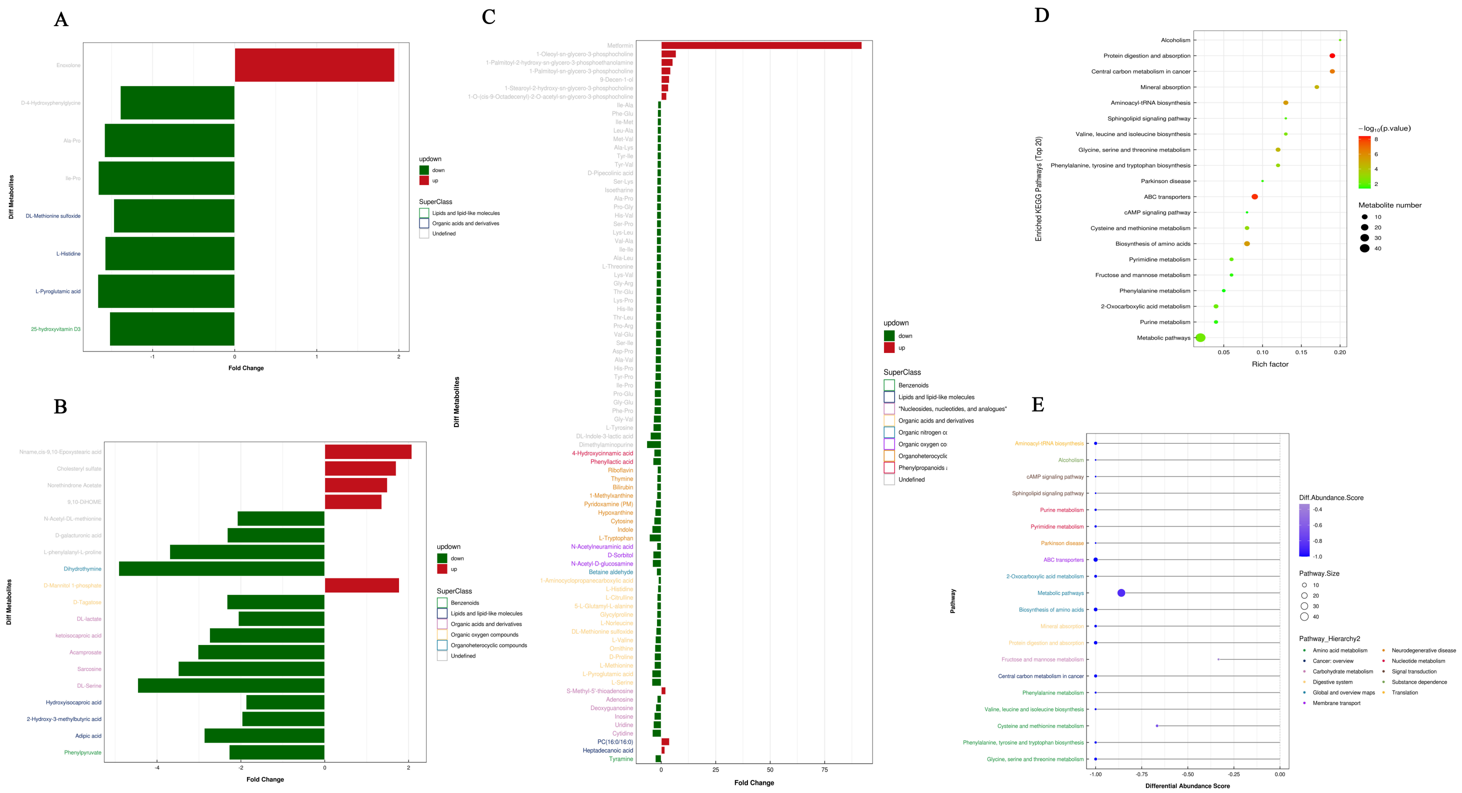

Supplemental Figure7 The metabolites difference and KEGG pathway enrichment analysis

PI: prefrailty group before receive prebiotic intervention; PM: prefrailty group before receive placebo intervention; FI: frailty group before receive prebiotic intervention; FM: frailty group before receive placebo intervention; PIA, PI group after receive prebiotic intervention. PMA, PM group after receive placebo intervention. FIA, FI group after receive prebiotic intervention. FMA, FM group after receive placebo intervention. A: Positive ion mode between group PMA and PIA, B: Anion ion mode between group FMA and FIA, C: Positive ion mode between group FMA and FIA, D: Bubble diagram of KEGG enrichment pathway between FMA and FIA, E: Differential abundance score plot of all differential metabolic pathways between FMA and FIA.

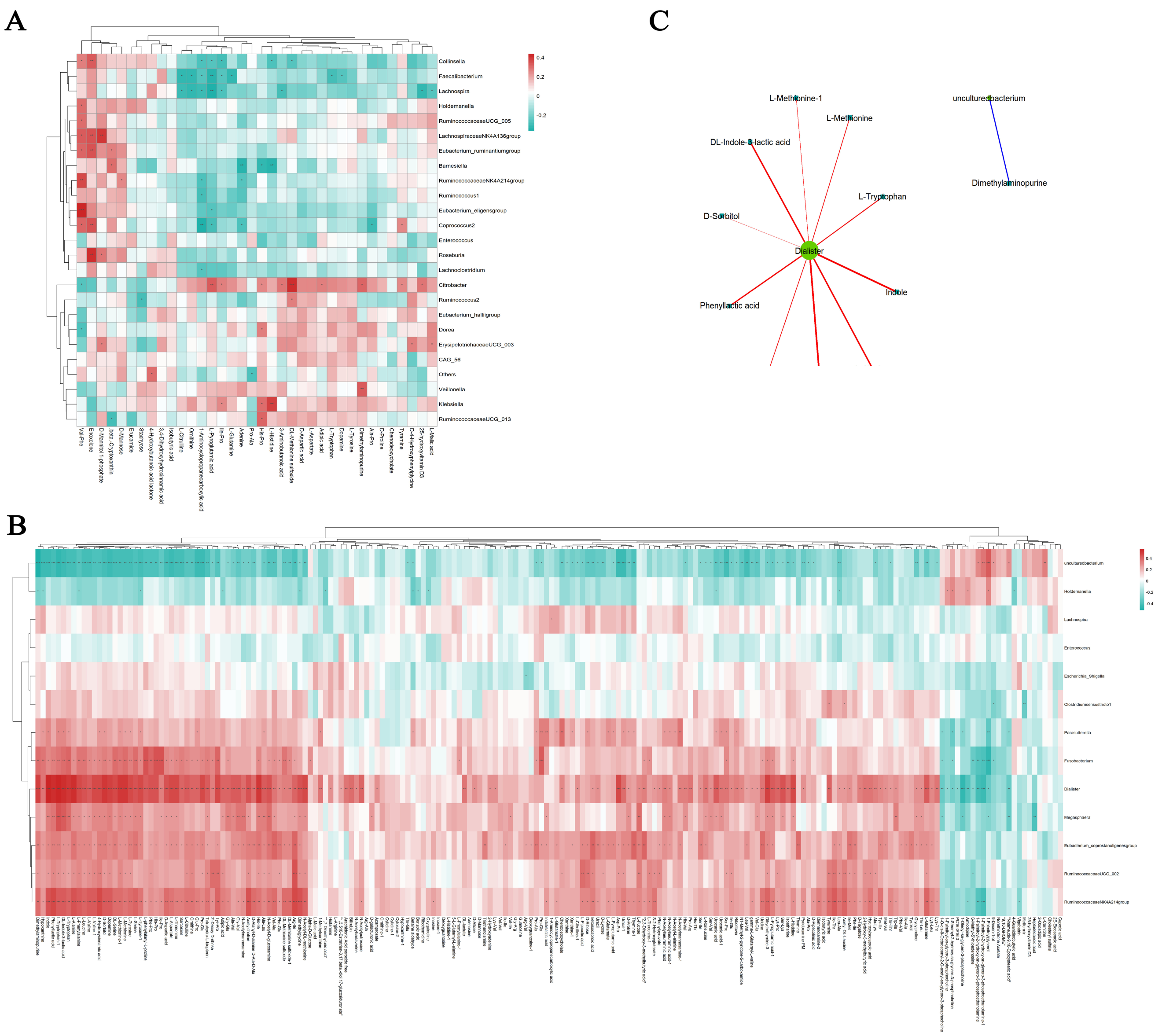

Supplemental Figure8 Correlation analysis of significant difference microbiota and metabolites hierarchical clustering heat map

PI: prefrailty group before receive prebiotic intervention; PM: prefrailty group before receive placebo intervention; FI: frailty group before receive prebiotic intervention; FM: frailty group before receive placebo intervention; PIA, PI group after receive prebiotic intervention. PMA, PM group after receive placebo intervention. FIA, FI group after receive prebiotic intervention. FMA, FM group after receive placebo intervention. A: PIA vs PMA, B: FIA vs FMA, C: network map between FIA and FMA

Supplementary Table 1 Intake of food and major nutrients among groups with different frailty states [M(Q1,Q3)]

	N (n=100)	P (n=100)	F (n=100)	*Z*	*P*
Staple Food (g)	330.35(227.10, 445.85)	318.35(240.83, 462.25)	353.35(230.83, 536.68)	1.834	0.400
Vegetables (g)	149.15(75.80, 205.02)	135.00(64.15, 202.90)	151.65(83.30, 285.85)	4.601	0.100
Fruit (g)	66.70(0.00, 166.70)	83.30(0.83, 150.00)	75.00(0.00, 200.00)	2.129	0.345
Eggs (g)	28.35(0.00, 50.00)	33.30(8.73, 50.00)	33.30(16.70, 66.70)	5.550	0.062
Meat (g)	16.70(0.00, 45.45)	16.70(0.00, 41.93)	16.70(0.00, 50.00)	1.440	0.487
Beans (g)	8.35(0.00, 45.83)	0.00(0.00, 33.30)	0.00(0.00, 59.18)	1.138	0.566
Milk (g)	83.30(0.00, 166.70)	83.30(0.00, 205.02)	166.70(0.00, 250.00) [a]	9.392	0.009
Oil (g)	13.30(6.70, 24.58)	13.30(6.70, 22.48)	13.30(6.70, 26.70)	2.090	0.352
Energy (kcal)	1537.30(1126.00, 1893.45)	1490.70(1270.35, 1788.30)	1589.75(1199.40, 2149.98)	2.017	0.365
Protein (g)	44.90(36.30, 59.28)	44.80(38.38, 57.03)	51.35(37.53, 69.33)	5.077	0.079
Fat (g)	38.30(24.25, 57.48)	35.00(23.93, 49.85)	40.80(28.05, 63.23)	4.974	0.083
Carbohydrates (g)	254.25(172.33, 322.48)	244.75(189.20, 300.38)	261.25(178.63, 373.78)	1.357	0.507
Dietary Fiber (g)	7.80(5.32, 11.98)	7.65(5.00, 11.28)	9.00(4.43, 15.45)	1.861	0.394

Compared with N, [a] $P < 0.05$; Compared with P, [b]$P < 0.05$.

Supplementary table 2 Baseline comparison of frailty index, scales and body composition in prefrail group

	PM (n=50)	PI (n=50)	$t/Z/\chi 2$	P
Age (years) [M(Q1,Q3)]	73.00（68.75, 80.25）	73.00（71.00, 77.00）	-0.19	0.85
Gender (male, %)	17（34%）	23（46%）	1.5	0.22
Shrinking（weight loss）	4（8%）	8（16%）	1.52	0.36
Low activity（n, %）	19（38%）	13（26%）	1.65	0.28
Slowness（walking time）（s, $\bar{x}\pm s$）	5.72±1.03	5.90±1.28	-0.77	0.41
Weakness（grip strength）（kg，$\bar{x}\pm s$）	19.21±6.70	21.11±7.24	-1.37	0.18
Poor endurance [M(Q1,Q3)]	0.00（0.00, 2.00）	0.00（0.00, 2.00）	-1.21	0.29
ADL（$\bar{x}\pm s$）	97.40±5.82	97.90±3.92	-0.5	0.62
SAS（$\bar{x}\pm s$）	32.01±7.99	30.24±8.32	1.12	0.27
GDS（$\bar{x}\pm s$）	7.68±4.64	6.96±3.84	0.85	0.40
PAC-QOL [M(Q1,Q3)]	10（0,19）	13.00（3.75, 23.00）	-0.89	0.37
PSQI（$\bar{x}\pm s$）	7.24±2.96	7.04±3.53	-0.71	0.48
Weight（kg，$\bar{x}\pm s$）	61.93±10.29	63.88±10.49	-0.94	0.35
BMI（kg/m^2，$\bar{x}\pm s$）	21.80±3.67	22.35±4.36	-0.69	0.49
Fat percentage（$\bar{x}\pm s$）	25.01±10.10	25.02±10.45	-0.01	1.00
Muscle mass（$\bar{x}\pm s$）	43.05±3.60	44.13±3.59	-1.49	0.14
Bone mass（kg, $\bar{x}\pm s$）	2.67±0.28	2.74±0.33	-1.14	0.26
Visceral fat grade（$\bar{x}\pm s$）	6.77±3.58	6.79±3.51	-0.02	0.98
Basal metabolism（kcal, $\bar{x}\pm s$）	1291.72±121.93	1333.22±127.78	-1.66	0.10
Body moisture rate（$\bar{x}\pm s$）	52.97±7.43	53.91±8.76	-0.58	0.57

Supplementary table 3 Baseline comparison of major dietary components and nutrient intake in prefrailty group[M(Q1,Q3)]

	PM (n=50)	PI (n=50)	Z	P
Staple Food (g)	313.3 (245, 438.35)	320 (233.3, 430)	-0.176	0.860
Vegetables (g)	133.3 (60, 206.65)	136.7 (66.7, 201.7)	-0.168	0.867
Fruit (g)	100 (25, 158.35)	66.7 (0, 150)	-0.804	0.421
Eggs (g)	33.3 (11.65, 50)	41.7 (0, 58.3)	-0.687	0.492
Meat (g)	10 (0, 39.15)	16.7 (0, 42)	-1.095	0.274
Beans (g)	0 (0, 33.3)	0 (0, 45)	-0.566	0.571
Milk (g)	83.3 (0, 208.35)	83.3 (0, 206.7)	-0.407	0.684
Oil (g)	6.7 (0, 20)	16.7 (10, 25)	-2.892	0.004
Energy (kcal)	1470.8 (1272.9, 1796.9)	1547.3 (1266.7, 1780.8)	-0.394	0.693
Protein (g)	45 (39.2, 57)	44.6 (36.8, 57.2)	-0.5	0.617
Fat (g)	33.3 (21.05, 44.7)	35.6 (29.5, 51.3)	-1.062	0.288
Carbohydrates (g)	233.3 (183.95, 336.1)	251 (190.4, 298.8)	-0.144	0.885
Dietary Fiber (g)	8 (5.8, 11.6)	6.5 (4.8, 11.4)	-0.785	0.432

Supplementary table 4 Baseline comparison of liver and kidney function and cytokines in prefrailty group

	PM (n=50)	PI(n=50)	*t/Z*	*P*
Albumin/globulin	1.31±0.24	1.29±0.17	0.384	0.701
Albumin (g/L)	43.89±8.12	41.71±7.79 b	1.37	0.174
Alkaline phosphatase (U/L)	90.72±39.01	83.66±28.74	1.03	0.305
Alanine aminotransferase (ALT, U/L)	22.46±11.99	17.16±14.29	2.009	0.047
Aspartate Aminotransferase (AST, U/L)	27.14±13.91	23.22±10.59	1.586	0.116
AST/ALT	1.48±0.33	1.42±0.44	0.775	0.44
Urea (mmol/L)	6.10±2.4	7.09±3.23	-1.752	0.083
Creatinine (μmol/L)	78.06±26.61	89.52±49.66	-1.438	0.154
Cystatin C (mg/L)	1.43±0.38	1.52±0.53	-1.054	0.295
Direct bilirubin (μmol/L)	4.40（2.9,6.05）	4.05（2.98,6.13）	-0.576	0.565
Globulin (g/L)	33.81±9.63	29.08±7.35	2.763	0.007
Gamma-glutamyltransferase (U/L)	13(7.00,26.50)	19.5(11.00,36.25)	-2.069	0.038
Indirect bilirubin (μmol/L)	6.55(4.35,9.10)	6.25(3.28,10.85)	-0.065	0.948
Total bilirubin (μmol/L)	11.20(8.38,15.03)	10.75(6.25,17.15)	-0.458	0.647
Total protein (g/L)	75.70±15.06	70.79±13.77	1.702	0.092
Uric acid (μmol/L)	321.98±143.46	335.2±133.61	-0.477	0.635
IL-17(pg/ml)	29.45（17.25，48.93）	30.12（15.99，52.09）	-0.152	0.879
γ-IFN（pg/ml）	2.82（2.21，4.05）	4.05（3.44，5.06）	-4.128	＜0.001
IGF-1(ng/ml)	113.56（79.32，148.53）	102.42（79.08，154.86）	-0.031	0.975

Supplementary table 5 Baseline comparison of frailty index, scales and body composition in frailty group [M(Q1,Q3)]

	FM (n=50)	FI (n=50)	*t/Z/χ2*	*P*
Age (years) [M(Q1,Q3)]	79.00（73.75,81.00）	77.00（73.00,81.50）	-0.80	0.42
Gender (male, %)	19(38%)	20(40%)	0.84	0.47
Shrinking（weight loss）	7(14%)	12(24%）	2.92	0.23
Low activity（n, %)	26(52%)	26(52%)	0.11	1.00
Slowness（walking time）（**s,** $\bar{x}$±*s*）	7.94±1.79	8.23±2.47	0.47	0.50
Weakness（grip strength）（**kg**，$\bar{x}$±*s*）	15.64±5.30	15.64±6.29	0.00	1.00
Poor endurance **[M(Q1,Q3)]**	2.42±0.97	2.14±1.17	1.64	0.20
ADL（$\bar{x}$±*s*）	96.00±6.06	96.63±4.72	0.34	0.56
SAS（$\bar{x}$±*s*）	1.00（1.00，1.00）	1.00（1.00，1.00）	1.01	1.00
GDS（$\bar{x}$±*s*）	1.00（1.00，1.00）	1.00（1.00，2.00）	5.98	0.05
PAC-QOL **[M(Q1,Q3)]**	12.50（4.00,19.00）	13.00（6.00,26.00）	-1.40	0.16
PSQI（$\bar{x}$±*s*）	7.00（4.00,10.25	8.00（5.50，10.00）	-0.20	0.84
Weight（**kg**，$\bar{x}$±*s*）	59.37±13.14	61.52±14.92	0.58	0.45
BMI（**kg/m^2**，$\bar{x}$±*s*）	20.83±4.66	21.53±5.24	0.50	0.48
Fat percentage（$\bar{x}$±*s*）	21.70±11.52	25.52±10.91	2.86	0.09
Muscle mass（$\bar{x}$±*s*）	42.73±7.23	41.84±7.27	0.37	0.54
Bone mass（**kg,** $\bar{x}$±*s*）	2.60±0.47	2.63±0.51	0.06	0.80
Visceral fat grade（$\bar{x}$±*s*）	5.79±3.94	6.86±3.86	1.85	0.18
Basal metabolism（**kcal,** $\bar{x}$±*s*）	1251.56±258.65	1274.76±236.96	0.22	0.64
Body moisture rate（$\bar{x}$±*s*）	54.70±12.53	50.58±9.97	3.26	0.07

Supplementary table 6 Baseline comparison of major dietary components and nutrient intake in frailty group[M(Q1,Q3)]

	FM (n=50)	FI (n=50)	*Z*	*P*
Staple Food (g)	224.55(310.75, 442.95)	269.2(400, 581.75)	-1.958	0.050
Vegetables (g)	77.08(146.65, 244.58)	87.48(200, 395.83)	-1.372	0.170
Fruit (g)	0(66.7, 179.15)	33.3(121.15, 266.7)	-1.409	0.159
Eggs (g)	16.7(37.5, 59.58)	1.68(33.3, 74.2)	-0.244	0.807
Meat (g)	0(25, 47.93)	0(16.7, 64.58)	-0.768	0.443
Beans (g)	0(0, 51.28)	0(0, 65.03)	-0.322	0.748
Milk (g)	0(133.3, 247.5)	0(166.7, 250)	-0.962	0.336
Oil (g)	10(16.7, 32.05)	0.83(13.3, 23.3)	-1.816	0.069
Energy (kcal)	1169.83(1477.85, 1928.95)	1278.35(1718.15, 3319.5)	-1.509	0.131
Protein (g)	37.53(49.1, 66.35)	37.53(56, 100.18)	-1.333	0.183
Fat (g)	28.05(37.4, 59.85)	28.25(43.4, 66.15)	-0.692	0.489
Carbohydrates (g)	178.63(226, 327.53)	181.63(296.05, 480.88)	-1.892	0.059
Dietary Fiber (g)	9（4.0, 11.98）	5.5(9.55, 19.2)	-1.173	0.241

Supplementary table 7 Baseline comparison of liver and kidney function and cytokines in frailty group

	FM(n=50)	FI(n=50)	*t/Z*	*P*
Albumin/globulin	1.28±0.19	1.29±0.15	-0.35	0.727
Albumin (g/L)	39.3±9.23 c	39.04±7.28 d	0.155	0.877
Alkaline phosphatase (U/L)	95.12±35.94 c	85.52±47.40	1.141	0.257
Alanine aminotransferase (ALT, U/L)	16.76±17.18	16.00±15.19	0.234	0.815
Aspartate Aminotransferase (AST, U/L)	20.56±16.03 c	21.08±11.01	-0.189	0.85
AST/ALT	1.51±0.48	1.46±0.40	0.631	0.529
Urea (mmol/L)	6.18±2.61	5.57±2.01	1.311	0.193
Creatinine (μmol/L)	65.98±28.72	65.92±21.46	0.012	0.991
Cystatin C (mg/L)	1.35±0.45	1.14±0.30	2.813	0.006
Direct bilirubin (μmol/L)	3.80(2.35,4.95)	3.95(2.88,4.83)	-0.462	0.644
Globulin (g/L)	29.62±9.86	29.29±7.35	0.191	0.849
Gamma-glutamyltransferase (U/L)	17.00(12.00,30.00)_	17(8.50,33.25)	-0.266	0.791
Indirect bilirubin (μmol/L)	5.30(3.10,9.18)	5.55(3.10,8.58)	-0.314	0.754
Total bilirubin (μmol/L)	8.10(4.85,13.60)	9.55(6.43,13.33)	-0.662	0.508
Total protein (g/L)	67.92±17.84	68.33±13.67	-0.129	0.898
Uric acid (μmol/L)	323.3±149.39	332.54±123.52	-0.337	0.737
IL-17(pg/ml)	31.08（19.02，50.49）	43.49（23.07，70.82）	-1.761	0.078
γ-IFN（pg/ml)	10.92（8.09，15.44）	9.98（8.71，11.87）	-0.587	0.558
IGF-1(ng/ml)	67.46（58.84，75.85）	65.69（59.70，77.17）	-0.393	0.694

The specific experimental methodology section is as follows:

16S sequencing

1 Method 1.1 Extraction of genome DNA Total genome DNA from samples was extracted using CTAB/SDS method. DNA concentration and purity was monitored on 1% agarosegels. According to the concentration, DNA was diluted to 1ng/µl using sterile water. 1.2 Amplicon Generation Primer： Amplified region Primer V3-V4 341F： CCTAYGGGRBGCASCAG 806R： GGACTACNNGGGTATCTAAT V4 515F： GTGCCAGCMGCCGCGGTAA 806R GGACTACH VG G GTWTCTAAT 16S rRNA genes were amplified used the specific primer with the barcode. All PCR reactions were carried out in 30µL reactions with 15µL of Phusion®High-Fidelity PCR Master Mix (New England Biolabs); 0.2µM of forward and reverse primers, and about 10 ng template DNA. Thermal cycling consisted of initial denaturation at 98 ℃ for 1 min, followed by 30 cycles of denaturation at 98℃ for 10 s, annealing at 50 ℃ for 30 s, and elongation at 72 ℃ for 60 s. Finally 72 ℃ for 5 min. 1.3 PCR Products quantification and qualification Mix same volume of 1X loading buffer (contained SYB green) with PCR products and operate electrophoresis on 2% agarose gel fordetection. Samples with bright main strip between 400-450bp were chosen for further experiments. 1.4 PCR Products Mixing and Purification PCR products was mixed in equidensity ratios. Then, mixture PCR products was purified with AxyPrepDNA Gel Extraction Kit (AXYGEN). 1.5 Library preparation and sequencing Sequencing libraries were generated using NEB Next®Ultra™DNA Library Prep Kit for Illumina (NEB, USA) followingmanufacturer's recommendations and index codes were added. The library quality was assessed on the Qubit@ 2.0 Fluorometer (Thermo Scientific) and Agilent Bioanalyzer 2100 system. At last, the library was sequenced on an Illumina Novaseq 6000 platform and 250bp/300bp paired-end reads were generated. 2 Data analysis 2.1 Paired-end reads assemblies Paired-end reads from the original DNA fragments were merged using FLASH, a very fast and accurate analysis tool, which was designed to merge paired-end reads when at least some of the reads overlap the read generated from the opposite end of the same DNA fragment. Paired-end reads was assigned to each sample according to the unique barcodes. 2.2 OTU cluster and Species annotation Sequences analysis were performed by UPARSE software package using the UPARSE-OTU and UPARSE-OTUref algorithms. In-house Perl scripts were used to analyze alpha (within samples) and beta (among samples) diversity. Sequences with ≥97% similarity were assigned to the same OTUs. We pick a representative sequences for each OTU and use the RDP classifier to annotate taxonomic information for each representative sequence. In order to compute Alpha Diversity, we rarify the OTU table and calculate three metrics: Chao1 estimates the species abundance; Observed Species estimates the amount of unique OTUs found in each sample, and Shannon index. Rarefaction curves were generated based on these three metrics. 2.3 Phylogenics distance and community distribution Graphical representation of the relative abundance of bacterial diversity from phylum to species can be visualized using Krona chart. Cluster analysis was preceded by principal component analysis (PCA), which was applied to reduce the dimension of the original variables using the QIIME software package. QIIME calculates both weighted and unweighted unifrac distance, which are phylogenetic measures of beta diversity. We used unweighted unifrac distance for Principal Coordinate Analysis (PCoA) and Unweighted Pair Group Method with Arithmetic mean (UPGMA) Clustering. PCoA helps to get principal coordinates and visualize them from complex, multidimensional data. It takes a

transformation from a distance matrix to a new set of orthogonal axes. By which the maximum variation factor is demonstrated by first principal coordinate, and the second maximum one by the second principal coordinate, and so on. UPGMA Clustering is a type of hierarchical clustering method using average linkage and can be used to interpret the distance matrix. 2.4 Statistical analysis To confirm differences in the abundances of individual taxonomy between the two groups, STAMP software was utilized. LEfSe was used for the quantitative analysis of biomarkers within different groups. This method was designed to analyze data in which the number of species is much higher than the number of samples and to provide biological class explanations to establish statistical significance, biological consistency, and effect-size estimation of predicted biomarkers. To identify differences of microbial communities between the two groups, ANOSIM and ADONIS were performed based on the Bray-Curtis dissimilarity distance matrices

Untargeted metabolomics

Metabolite Extractions To extract metabolites from the samples, 1 mL cold extraction solvent methanol/acetonitrile/H2O (2:2:1, v/v/v) was added to 80 mg sample, and adequately vortexed. After vortexing, the samples were incubated on ice for 20 minutes, and then centrifuged at 14,000 g for 20 minutes at 4°C, the supernatant was collected and flowed through a 96-well protein precipitation plate, and then the elution was collected and dried in a vacuum centrifuge at 4°C. For LC-MS analysis, the samples were re-dissolved in 100 μL acetonitrile/water (1:1, v/v) solvent and transferred to LC vials.

LC-MS Analysis For untargeted metabolomics of polar metabolites, extracts were analyzed using a quadrupole time-of-flight mass spectrometer (Sciex TripleTOF 6600) coupled to hydrophilic interaction chromatography via electrospray ionization in Shanghai Applied Protein Technology Co., Ltd. LC separation was on a ACQUIY UPLC BEH Amide column (2.1 mm × 100 mm, 1.7μm particle size (waters, Ireland) using a gradient of solvent A (25 mM ammonium acetate and 25 mM ammonium hydroxide in water) solvent B (acetonitrile). The gradient was 85% B for 1 min and was linearly reduced to 65% in 11 min, and then was reduced to 40% in 0.1 min and kept for 4 min, and then increased to 85% in 0.1 min, with a 5 min re-equilibration period employed. Flow rate was 0.4 mL/minute, column temperature was 25 °C, auto sampler temperature was 5°C, and injection volume was 2 μL. The mass spectrometer was operated in both negative ion and positive ionizations mode. The ESI source conditions were set as follows: Ion Source Gas1 (Gas1) as 60, Ion Source Gas2 (Gas2) as 60, curtain gas (CUR) as 30, source temperature: 600℃, IonSpray Voltage Floating (ISVF) ± 5500 V. In MS acquisition, the instrument was set to acquire over the m/z range 60-1000 Da, and the accumulation time for TOF MS scan was set at 0.20 s/spectra. In auto MS/MS acquisition, the instrument was set to acquire over the m/z range 25-1000 Da, and the accumulation time for product ion scan was set at 0.05 s/spectra. The product ion scan is acquired using information dependent acquisition (IDA) with high sensitivity mode selected. The parameters were set as follows: the collision energy (CE) was fixed at 35 V with ± 15 eV; declustering potential (DP), 60 V (+) and −60 V (−); exclude isotopes within 4 Da, candidate ions to monitor per cycle: 10. Data Analysis The raw MS data (wiff.scan files) were converted to MzXML files using ProteoWizard MSConvert before importing into freely available XCMS software. For peak picking, the following parameters were used: centWave m/z = 25 ppm, peakwidth = c (10,

60), prefilter = c (10, 100). For peak grouping, bw = 5, mzwid = 0.025, minfrac = 0.5 were used. In the extracted ion features, only the variables having more than 50% of the nonzero measurement values in at least one group were kept. Compound identification of metabolites by MS/MS spectra with an in-house database established with available authentic standards. After normalized to total peak intensity, the processed data were uploaded into before importing into SIMCA-P (version 14.1, Umetrics, Umea, Sweden), where it was subjected to multivariate data analysis, including Paretoscaled principal component analysis (PCA) and orthogonal partial leastsquares discriminant analysis (OPLS-DA). The 7-fold cross-validation and response permutation testing were used to evaluate the robustness of the model. The variable importance in the projection (VIP) value of each variable in the OPLS-DA model was calculated to indicate its contribution to the classification. Significance was determined using an unpaired Student's t test. VIP value >1 and $p<0.05$ was considered as statistically significant. Bioinformatics Analysis For KEGG pathway annotation, the metabolites were blasted against the online Kyoto Encyclopedia of Genes and Genomes (KEGG) database to retrieve their COs and were subsequently mapped to pathways in KEGG11. The corresponding KEGG pathways were extracted. To further explore the impact of differentially expressed metabolites, enrichment analysis was performed. KEGG pathway enrichment analyses were applied based on the Fisher' exact test, considering the whole metabolites of each pathway as background dataset. And only pathways with p-values under a threshold of 0.05 were considered as significant changed pathways. For hierarchical clustering, Cluster 3.0 (http://bonsai.hgc.jp/~mdehoon/software/cluster/software.htm) and the Java Treeview software (http://jtreeview.sourceforge.net) were used. Euclidean distance algorithm for similarity measure and average linkage clustering algorithm (clustering uses the centroids of the observations) for clustering were selected when performing hierarchical clustering. Heat map is often presented as a visual aid in addition to the dendrogram.
